# Nanosilver: An Old Antibacterial Agent with Great Promise in the Fight against Antibiotic Resistance

**DOI:** 10.3390/antibiotics12081264

**Published:** 2023-07-31

**Authors:** Kyra G. Kaiser, Victoire Delattre, Victoria J. Frost, Gregory W. Buck, Julianne V. Phu, Timea G. Fernandez, Ioana E. Pavel

**Affiliations:** 1 Department of Physical and Environmental Sciences, Texas A&M University Corpus Christi, 6300 Ocean Drive, Corpus Christi, TX 78412, USA; kkaiser@islander.tamucc.edu (K.G.K.); vdelattre@islander.tamucc.edu (V.D.); gregory.buck@tamucc.edu (G.W.B.); 2 Department of Life Sciences, Texas A&M University Corpus Christi, 6300 Ocean Drive, Corpus Christi, TX 78412, USA; 3 Department of Chemistry, Physics, Geology and the Environment, Winthrop University, 701 Oakland Avenue, Rock Hill, SC 29733, USA; frostv@winthrop.edu (V.J.F.); phuj2@mailbox.winthrop.edu (J.V.P.); 4 Department of Biology, Winthrop University, 701 Oakland Avenue, Rock Hill, SC 29733, USA

**Keywords:** nanosilver, antimicrobial applications, physicochemical properties, antibacterial mechanisms, synergy, antibiotic-resistant bacteria

## Abstract

Antibiotic resistance in bacteria is a major problem worldwide that costs 55 billion USD annually for extended hospitalization, resource utilization, and additional treatment expenditures in the United States. This review examines the roles and forms of silver (e.g., bulk Ag, silver salts (AgNO_3_), and colloidal Ag) from antiquity to the present, and its eventual incorporation as silver nanoparticles (AgNPs) in numerous antibacterial consumer products and biomedical applications. The AgNP fabrication methods, physicochemical properties, and antibacterial mechanisms in Gram-positive and Gram-negative bacterial models are covered. The emphasis is on the problematic ESKAPE pathogens and the antibiotic-resistant pathogens of the greatest human health concern according to the World Health Organization. This review delineates the differences between each bacterial model, the role of the physicochemical properties of AgNPs in the interaction with pathogens, and the subsequent damage of AgNPs and Ag^+^ released by AgNPs on structural cellular components. In closing, the processes of antibiotic resistance attainment and how novel AgNP–antibiotic conjugates may synergistically reduce the growth of antibiotic-resistant pathogens are presented in light of promising examples, where antibiotic efficacy alone is decreased.

## 1. Brief History of Silver (Ag) and Its Old Antimicrobial Applications

Silver has an extensive history because it has been used for multiple millennia spanning from the Before Common Era (B.C.E) to the present day (Table 1) [1,2,3]. This long-term use of silver stemmed from its anti-deteriorative activity and led to its recognition as the most important antimicrobial agent (i.e., antibacterial, antiviral, antiparasitic, and antifungal) that predated antibiotics [4,5,6,7,8,9].

*Before Common Era (B.C.E.):* The usage of silver for antibacterial purposes in B.C.E. civilizations was primarily through the preservation of food items in silver containers or the addition of a silver coin to beverages for long-term storage [1,9]. A fundamental discovery was the correlation between containers made of silver and food items remaining safe for consumption. Rulers of various nations (Alexander the Great and Cyrus the Great) only consumed water that was kept in silver vessels [1,6,10,11]. Even though bacteria were not known at that time, this connection between the slower decomposition of food with silver containers and cutlery contributed to the medical advancements seen today [8]. Due to the difficulty of interpretation of ancient texts, there are varying claims of the first recorded attempt of using silver as a therapeutic remedy. One of the oldest examples is a reference to silver as a therapeutic agent in 1500 B.C.E, during the Han dynasty in China [12]. Other recorded instances of medical procedures using silver include the 69 B.C.E. Roman Pharmacopeia describing a silver nitrate (AgNO_3_)-based medicine, the practice of Hippocrates using silver leaf for wound care, and an ancient medical system (Ayurveda) from India listing silver as a therapy component for multiple diseases [1,4,8,13].

*Pre-industrialization:* From B.C.E until the first Industrial Revolution in 1760, silver was used as a novel medical therapy for a broad spectrum of ailments (e.g., ulcers, wound infections, impure blood, heart palpitations, poor breath, epilepsy, and irritation) [1,14]. For example, Pliny the Elder, a Roman physician, described silver within his 79 C.E. (Common Era) book, *Natural History (Book XXXIII)*, as an effective healing agent within plasters and for wound closing [11,15]. Ambroise Paré, a French surgeon considered among the fathers of surgery, who served for multiple kings (Henry II, Francis II, Charles IX, and Henry III), used silver and other materials to construct ocular prosthetics [4,16]. Wealthier individuals in the Middle Ages, who regularly used silver utensils, overexposed themselves to silver and developed argyria (Figure 1), a rare skin condition that changes the color of skin, eyes, nails, and internal organs to a permanent blue-grey [1,17,18].

*During industrialization:* Key events such as the discovery of bacteria by Anton Leeuwenhoek in 1676 and the technological advancements associated with the Industrial Revolution in 1760 led to a transformation of medicine [2,19]. As antibiotics did not yet exist in the medical field, physicians used other agents (e.g., silver, mercury, copper, arsenic, and sulfur compounds) that were later deemed as beneficial, harmful, or entirely ineffective as therapeutic remedies [20]. Public attitudes toward health care were also drastically changed with the first public hospital, Bellevue Hospital, being officially established in New York City, in 1736 [21]. The concept of vaccination had its roots in 1796 through the work of physician Edward Jenner, who made the connection between patients who previously contracted cowpox and their immunity to smallpox [22]. He inoculated an 8-year-old boy with material from the cowpox lesions and concluded that the boy was protected from the illness [22]. This was the origin of transmittable protection, as in vaccination [23]. Vaccines were the most advanced medical agent, up until the 19th century, when the first antibiotic was discovered [24].

*Post industrialization:* In the 19th century, the physician Robert Koch made the claim that a certain bacterium can cause a specific disease. This led to Koch’s four postulates and the Germ Theory as it is seen today [25,26]. Following this, the physician Paul Ehrlich synthesized the first antimicrobial compound, salvarsan, in 1910 [24,27,28]. The physician scientist Alexander Fleming discovered the first true antibiotic to treat bacterial infections, penicillin, in 1928 [24,27,28]. Penicillin became available to the public later, in 1945 [29]. In this time, colloidal silver was being employed in hospital settings as an antibacterial agent, and silver salts were being administered to treat various infections and ailments (e.g., conjunctivitis, gonorrhea, gastroenteritis, syphilis, nicotine dependence, and mental illness) [8]. The German physician, Carl Siegmund Franz Credé, formulated in 1881 a 2% AgNO_3_ solution for neonatal conjunctivitis, which was so effective that it almost ended visual loss from the disease [8,30]. Other AgNO_3_ applications in the 1800s included therapies for burns, ulcers, compound fractures, and infections [1,31]. Physician Marion Sims employed to resolve the dilemma of post-delivery vesico-vaginal fistulas (when silk sutures failed) and administered silver-coated catheters during the healing period [1,31]. Colloidal silver (i.e., Ag particles suspended within a liquid) was first employed in 1891, by the surgeon B.C. Crede, as an antiseptic measure on wounds [1,12,32].

*Silver (Ag) forms*: As the scientific understanding of silver expanded over the course of history, the forms of Ag utilized also shifted (Figure 2). Initially, Ag was utilized in macro form (bulk Ag metal), when casting and forging household items (e.g., vessels, jewelry, and coins), or in atomic form (salt solutions of Ag^+^ ions), when treating wounds and other ailments. This was followed by the development and administration of micro- or nano-forms of Ag in water (colloidal Ag), as antibiotics were not yet available [8,28]. The first colloid of Ag was synthesized in the laboratory in 1889, by the chemist M. C. Lea [33,34]. In this redox reaction, citrate-capped AgNPs were intentionally created with dimensions of about 1–100 nanometers (nm) that changed their properties when compared to the Ag^+^ or bulk Ag forms [35]. However, the term nanotechnology was coined much later in 1974 by the Japanese professor Norio Taniguchi [36]. The first micro- and nanoparticles were visualized and characterized in 1981, after the invention of the first scanning tunneling microscope (STM). Nowadays, ionic silver (Ag^+^) and nanosilver (e.g., colloidal AgNPs) are the most emphasized forms of antimicrobial silver, which kill or inhibit the growth of microorganisms including pathogenic bacteria, viruses, and fungi, but cause little to no damage to the host.

## 2. Modern Antimicrobial Applications of Nanosilver

**Figure 3:**
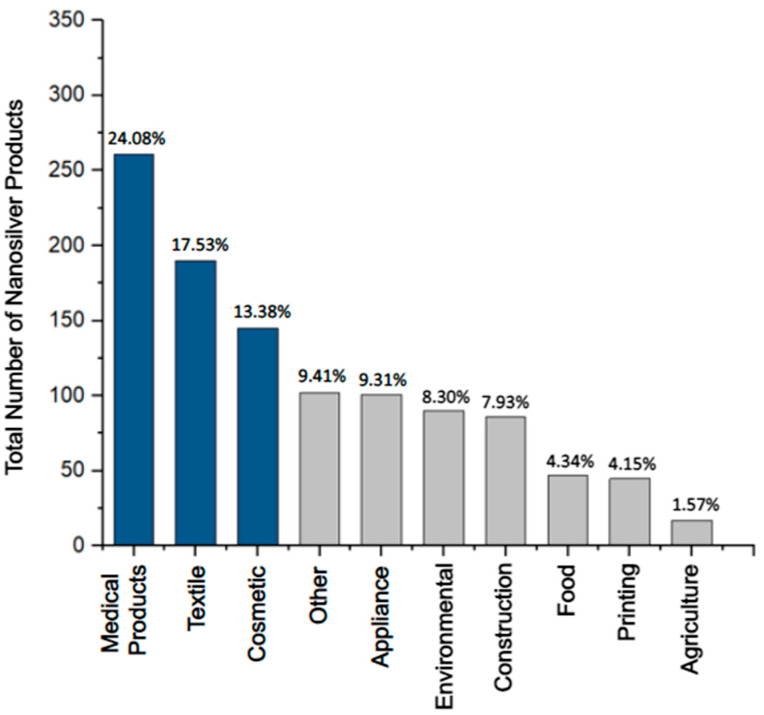
The most prevalent categories of consumer products containing nanosilver (U.S. FDA-approved and non-approved) make up 1084 registered products [46]. The top three sectors, the medical-, textile-, and cosmetics-related products (dark blue), are the most prominent categories, making up ~55% of the total number of consumer products containing nanosilver. The lesser seven categories (grey) make up ~45% of products.

The antimicrobial activity of nanosilver such as colloidal silver nanoparticles (AgNPs) is linked to its unique, size-related physicochemical properties such as the very large surface-to-volume ratios and the potential release of Ag^+^ ions from the nanosurface under favorable redox conditions. These properties are currently exploited in the manufacturing of everyday consumer products and other antimicrobial applications (Figure 3) [4,5].

*Antimicrobial consumer products*: In 2023, 5367 consumer products have been identified worldwide as containing nanomaterials by the manufacturer, and over 1000 of these products exploit the unique properties of nanosilver (e.g., antimicrobial, optical, and catalytical) [45,46]. Antimicrobial consumer products containing silver (Figure 3) can be found in the health (24.08%), textile (17.53%), cosmetic (13.38%), appliance (9.31%), environmental (8.30%), and construction (7.93%) sectors [46]. In the last few decades, the U.S. Food and Drug Administration (FDA) has approved many of these products containing antimicrobial Ag^+^ and nanosilver such as AgNPs. Examples include wound dressings, facial masks, textile fibers, sanitizers, coatings of surgical tools, dental implants, and urinary catheters (Table 2) [45,47].

“Silver wound dressings” represent the most web-searched (n = 2214—Table 2) and one of the most heavily used consumer products containing Ag in the medical sector. A large variety of U.S. FDA-approved (e.g., Silverlon, Aquacel Ag Advantage, and Acticoat) and non-approved wound dressings are offered through prescriptions as well as over the counter [48,49,50]. Silver-based wound dressings are used as both preventative and curative measures against bacterial infection of acute and chronic wounds. Textiles, the second most widespread application of nanosilver, have been used in many types of clothing (e.g., facemasks, socks, shirts, athletic wear, and towels) [45]. An illustrative example associated with nanosilver use is disinfectants in facemasks to prevent the spread of pathogens and the formation of malodor caused by bacterial colonies that inhabit the surface of the skin [51]. Manufacturers of cosmetics, the third largest sector, have employed nanosilver for the same antimicrobial benefits [52]. Nanosilver can be found in lotions, face masks, soaps, sunscreens, etc. [45].

Because the adverse effects of Ag on human health are not yet fully understood, concerns have been raised about the growing exposure to nanosilver during the manufacture or prolonged utilization of nanosilver-based consumer products [53]. Furthermore, the environmental health impacts of nanosilver remain under debate as nanosilver properties can change in the environment, leading to altered toxicity and stability [54,55,56]. The regulation of nanosilver-based consumer products has been compounded by the challenging task of tracking products that do not specify the nanomaterial as an ingredient, especially when in minute quantities, and by the product distribution under different brand names [57]. Nevertheless, the integration of nanosilver into consumer products continues to experience a vertiginous increase. An estimated 1000 tons of nanosilver is produced worldwide [58].

**Table 2:** The top applications of antimicrobial silver (Ag) together with illustrative products for each of the three major categories: health, textiles, and cosmetics. The vendor, the number of PubMed search results and selected key words, Ag form, [Ag quantities], product purpose, and U.S. FDA approval status are reported [49,59,60].

Product Type	Search Result	Vendor	[Ag] and Ag Form	Purpose	U.S. FDA Approval
Silver-based wound dressings	“silver wound dressing” n = 2214	Aquacel Ag Advantage—Convatec, Berkshire, England	1.2% *w*/*w*—“ionic silver”	To prevent and cure infection in acute or hard-to-heal wounds	YES
Ankle socks with silver	“silver textile” n = 1155	NanoSilver, Denmark, E.U.	Not Reported—“Nano-Silver”	To defend from pathogens	NO
Platinum silver nanocolloid cream	“silver cosmetic” n = 2292	DHC Skincare, Tokyo, Japan	Not Reported—“Nanosilver”	To eliminate bacteria in sweat	NO

*Other antimicrobial applications*: Lately, AgNPs and Ag^+^ have received increased attention due to their potential use in the fight against two major global health threats, namely antibiotic resistance and viral infections, where treatments are either limited or not available [61]. For instance, non-cytotoxic concentrations of AgNPs were reported to act against a broad spectrum of viruses of different families regardless of their tropism, clade, and resistance to antiretrovirals [61,62,63]. Relevant examples include HIV-1, hepatitis B (HBV), Tacaribe virus, herpes simplex virus, mpox, smallpox, H1N1 influenza A, respiratory syncytial viruses, vaccinia virus, and dengue virus (DENV). In these studies, AgNPs were found to bind specifically or nonspecifically to proteins in the envelope of virions and thereby deactivate them (virucidal activity). These target proteins are mainly responsible for the viral interaction with host cells [13,61,62,63]. During the pre-viral entry into host cells, AgNPs competitively attach to the cells and lyse the membrane of the virions (antiviral activity). In the case of the post-viral entry, AgNPs mainly inhibit the viral fusion with the cell membrane, and in several cases interfered with the stages of the viral replication cycle such as the synthesis of viral RNA (antiviral activity). At the molecular level, these mechanisms relied on the chemical interaction of AgNPs or Ag^+^ ions released by AgNPs with sulfur, nitrogen, or phosphorus-containing biomolecules including proteins and genetic material. Hence, AgNPs have multiple mechanisms of action, which suggests that resistance to AgNPs will be less likely to arise when compared to specific antiviral or antibiotic therapies [64,65,66].

The World Health Organization (WHO) has published a list of high-priority (first tier), antibiotic-resistant pathogens that present the greatest threat to human health. These include strains in the *Acinetobacter*, *Pseudomonas,* and various Enterobacteriaceae genera (*Klebsiella*, *Escherichia coli* (*E. coli*), *Serratia*, and *Proteus*) [67]. Most of these pathogens are Gram-negative strains that exhibit increased resistance when compared to the Gram-positive strains. Gram-negative bacteria have an outer membrane that contains lipopolysaccharide (LPS), which creates a permeability barrier against external, harmful factors [68]. For example, *Pseudomonas aeruginosa* (*P. aeruginosa*), a Gram-negative species that nanosilver-based products are commonly tested against, is listed as Priority 1 because the organism is CRITICAL due to its resistance to carbapenem antibiotics that are used as “last line” or “last resort” antibiotics [67,69]. Four of the six multi-drug-resistant (MDR) pathogens that are primarily responsible for infections originating from hospitalization are also Gram-negative bacteria, labeled as ESKAPE pathogens (*Enterococcus faecium (E. faecium*)*, Staphylococcus aureus (S. aureus*)*, Klebsiella pneumoniae (K. pneumoniae*)*, Acinetobacter baumannii (A. baumannii*)*, P. aeruginosa,* and *Enterobacter* species) [70]. Due to the imminent threat posed by Gram-negative and Gram-positive bacterial examples, this review mainly focuses on the potential use of AgNPs in the fight against antibiotic resistance, in these organisms.

## 3. Gram-Negative Bacteria (GNB) Versus Gram-Positive Bacteria (GPB) Models

There are multiple bacterial classifications based on the antigen susceptibility, biochemical reactions, phenotypic traits, and growth patterns, but before the bacterial strain is classified, the major group a species falls into must first be known [71]. The major groups of bacteria are separated by morphological properties including the Gram-stain result, cell shape (spherical-shaped cocci, rod-shaped bacilli, and spiral-shaped spirilla), method of motility (e.g., the presence of flagella), acid-fast result, endospore development, and presence of a capsule or inclusion bodies [71,72]. The variation in bacterial species dictates the cell’s vulnerability and resistance to extracellular substances, such as antibiotics or toxins that may be encountered in the environment. In particular, the membrane composition poses a substantial influence on the survival of bacteria because it serves as the first line of defense in a hostile environment, while also allowing selective permeability to nutrients and metabolites the cell needs to survive [73]. Gram-staining is utilized to differentiate bacteria into two major classification groups, Gram-positive and Gram-negative (with occasional Gram-variable strains). As outlined below, one of the main cell wall differences between the two groups is that Gram positive bacteria possess a thick peptidoglycan cell wall outside of the cytoplasmic membrane, while Gram-negative bacteria contain a thin layer of peptidoglycan between the cytoplasmic membrane and the outer membrane [73,74].

**Gram-Positive Bacteria (GPB)** (e.g., *E. faecium*, *S. aureus*, *Streptococcus pneumoniae* (*S. pneumoniae*), and *Bacillus brevis* (*B. brevis*)) exhibit a purple color upon Gram staining due to the primary applied crystal violet stain that adheres to the thick (20–80 nm) peptidoglycan cell wall external to the plasma membrane (Figure 4) [74,75,76]. The peptidoglycan consists of modified sugars, N-acetyl glucosamine (NAG), and N-acetyl muramic acid (NAM); cross-linking occurs with amino acid linkages between NAM residues. The peptidoglycan or murein provides cell stability, shape, resistance to osmotic pressure, physical protection from the environment, and additional defense from consistent remodeling in environmental adaptation [77,78]. This polymer layer contains teichoic acids, which attach to the peptidoglycan covalently through phosphodiester linkages (wall teichoic acid (WTA)) and are anchored to the membrane through a glycolipid on the plasma membrane, while elongating through the peptidoglycan (lipoteichoic acid (LTA)) [73,79]. Teichoic acids contribute to the overall hydrophobicity and the cell’s surface charge (e.g., −86 mV for *B. brevis*) because they possess metal cations, which promote attraction to negative phosphate groups and assist in homeostasis and protection of the cell [80,81,82,83]. Even though most MDR pathogens (ESKAPE) are not GPB, there is still concern for infections caused by GPB strains.

**Gram-Negative bacteria (GNB)** (e.g., *P. aeruginosa*, *K. pneumoniae*, *Vibrio vulnificus* (*V. vulnificus*), *A. baumannii*, *E. coli*, and other *Enterobacter* species) [83,84,85,86] exhibit a pink or red color due to the absence of crystal violet, when the Gram staining process adds safranin as a counterstain [74,75]. Peptidoglycan is present in GNB, but in a thin layer (5–10 nm) (Figure 5) that is sometimes a single layer (e.g., up to 80% of peptidoglycan is present as a monolayer in *E. coli*) [74,87]. To compensate for the lower peptidoglycan content, GNB bear an outer membrane (7.5–10 nm thick) that holds lipopolysaccharides (LPS) in most species, which are exclusive to GNB cells [74,88,89]. Like peptidoglycan, LPS is primarily responsible for the structural integrity of the cell [86]. Unlike the phospholipid bilayer structure of the plasma membrane, the outer membrane is asymmetrical, with LPS on the outer leaflet of the outer membrane and phospholipids facing the inner peptidoglycan layer and cytoplasm [90,91]. Lipid A or endotoxin, one of the three components of LPS (along with hydrophilic polysaccharide core and branched O antigen), contributes to the higher potency of stimulating a host immune response and varies in structure between species [86,91]. LPS on the outer membrane surface lowers the permeability to lipophilic compounds (e.g., certain antibiotics such as fluoroquinolones, macrolides, tigecycline, and lincosamides) by serving as a barrier to the extracellular space [68,92]. The structure of LPS is very dense, as polysaccharide chains extend outward and can pack closely together through ionic interactions between the anionic phosphate groups and cations present [93,94]. These interactions enhance the barrier abilities of LPS.

Based on the specialized membrane structure of GNB that increases the permeability barrier, GNB strains typically exhibit higher antibiotic resistance than their GPB counterparts. The dilemma of antibiotic resistance is crucial as the CDC approximated that the expenses related to antibiotic resistance total to 55 billion USD annually in the U.S. alone, and 35 billion USD for loss of productivity [95,96]. These structural differences of the membrane alter the possible interactions with antimicrobial AgNPs, which also differ depending on the physicochemical properties of AgNPs.

## 4. Silver Nanoparticle (AgNP) Models

*Fabrication of AgNPs:* There are two main categories of nanofabrication methods: “top-down” and “bottom-up” (Figure 6). Each fabrication approach has its unique set of advantages and disadvantages, and the two can be intertwined (hybrid methods) to lead to a desired set of PCC properties for a nanomaterial [97]. The “top-down” method breaks down a bulk material into powder or fragments that are further reduced to NPs by physical or chemical processes [97]. The “bottom-up” method does the opposite; atoms or molecules react under chemical, biological, and physical conditions to form clusters or nuclei that assemble into NPs [97]. For example, in a ‘’top-down’’ approach, evaporation of a solid Ag source (bulk), which is first melted through the Joule heating of the resistive boat hosting the metal source, can produce Ag atoms and Ag nanoclusters (thermal evaporation) [98,99]. However, in a “bottom-up” procedure, these evaporated atoms of Ag can travel to a solid substrate to form a thin layer (film) of Ag through nucleation under high-vacuum (10^−6^ torr) conditions (chemical vapor deposition) [100,101].

Chemical and biological processes are mainly used in “bottom-up” fabrications because they require the manipulation of weaker intermolecular interactions [97]. In contrast, physical approaches are typically preferred in ‘’top-down’’ fabrications, when strong covalent bonds must be broken [97]. Laser ablation and evaporation–condensation are among the most common physical processes used to produce AgNPs [114]. A PubMed search using the words “laser ablation silver nanoparticles” and “evaporation silver nanoparticles” led to n = 345 and n = 234 articles (Table 3), respectively. AgNPs fabricated by physical processes (Table 3) have a narrow size distribution and less risk of contamination by solvents or other reagents than in chemical processes [115,116]. However, the high energy consumption and modest yield lower the cost-efficiency of the physical fabrication methods [116]. In recent years, biological processes have been developed to replace chemical and physical processes that are expensive or use hazardous substances (Table 3). In the biological approaches, harmful reducing and capping agents are substituted with biocompatible compounds such as plant extracts, bacteria, fungi, and enzymes [116,117]. For example, bacteria from Zarshouran gold mines (Iran) that are tolerant to Ag^+^ ions were isolated (*Bacillus* ROM6) and utilized in the synthesis of spherical AgNPs of 25 nm in diameter, with 90% efficiency and using less than 0.9 g L^−1^ of AgNO_3_ [118]. Roughly spherical AgNPs of 4 nm in diameter were fabricated using 30 g of honey irradiated with gamma radiation (5 kGy) as reducing and capping agents [119]. These honey-capped AgNPs were found to kill both GNB and GPB (minimum inhibitory concentration (MIC) ranging from 1.69 to 6.25 µg mL^−1^) but were more efficient in GNB [119]. These new kinds of colloidal AgNPs are eco-friendly and biocompatible with medical and environmental applications [120,121]. However, biological processes lead to the formation of less homogeneous AgNPs, which require extensive or costly purification [122].

Chemical syntheses continue to prevail over physical and biological techniques in the fabrication of colloidal AgNPs [123]. This is despite the hazardous nature of most chemical reagents and the eco-unfriendly attributes of numerous chemical methods. A PubMed search using the words “silver nanoparticles chemical fabrication“ and “silver salt to silver nanoparticles” revealed n = 1459 and n = 690 publications, respectively. The reduction of metal salts is the most used ”bottom-up”’’ fabrication for colloidal AgNPs [114]. Chemical approaches have several key components: a silver precursor such as a silver salt containing Ag^+^ ions, a reducing agent for the Ag^+^ ions, a solvent, a capping agent, and an optional functionalization agent for the nanosurface [116]. In the redox reaction, Ag^+^ ions are reduced to Ag^0^ in solution, which then form clusters through the nucleation of atoms (Figure 7). These clusters continue to grow into AgNPs that stabilize with the help of a capping agent. Capping agents are in general used to control the stability, size, shape, reactivity, and solubility of AgNPs [124]. A comprehensive review of chemical fabrication methods [126] shows that most colloidal AgNPs have a negative surface charge and are produced using AgNO_3_ as a metal precursor (>80% of the n = 690 reviewed articles) and reducing agents such as sodium borohydride (23%, the Creighton method) or trisodium citrate (10%, the Lee Meisel method) before subsequent functionalization. These low-cost reducing agents lead to high reaction yields and ease the functionalization process [116]. Water (>80%) is the preferred solvent due to its low environmental and biological impact. Citrate from trisodium citrate is the most commonly used capping agent (50%) and is followed by polyvinyl pyrrolidone (PVP, 18%), cetyltrimethylammonium bromide (CTAB), amines, amides, and fatty acids [127]. In addition, AgNPs can be functionalized to increase stability and prevent agglomeration [128]. AgNP functionalization is particularly useful in therapeutic and medical diagnostic applications [128]. For example, the antibacterial properties of core AgNPs can be further boosted through functionalization with antibiotics (e.g., streptomycin) or non-antibiotic agents (e.g., other antibacterial agents such as chitosan and polyphenol biomolecules) [129,130]. AgNPs functionalized with streptomycin, an antibiotic medication used to treat a plethora of bacterial infections, exhibited increased antibacterial activity against *Staphylococcus aureus* (MRSA) when compared to AgNPs or antibiotics alone [129]. AgNPs conjugated to cefadroxil, an antibacterial drug of strong activities against various bacterial strains, enhanced the antibacterial potential of cefadroxil alone up to twofold against *S. aureus* [131]. AgNPs functionalized with antibacterial chitosan and seaweed-derived polyphenols demonstrated superior, synergistic, antibacterial activity against both GNB and GPB strains (*E. coli*, *Proteus*, *Salmonella*, and *Bacillus cereus*) when compared to the unfunctionalized AgNPs [130]. Natural products as alternative antimicrobial compounds have also been investigated to circumvent the possible side-effects of existing antibiotics. For example, researchers determined that antibacterial compounds capable of perforating the barrier of antibacterial resistance can be found in the epicarp of the yellow Malaysian rambutan fruit (*Nephelium lappaceum*) [132]. Initial screening of these crude extracts by Kirby–Bauer disk diffusion assays using different solvents showed promising antibacterial effects against *B. subtilis*, *P. aeruginosa*, *S. enterica*, MRSA, and *S. pyogenes* [132]. Ethyl acetate or acetone fractions subjected to chemical profiling by common separation methods identified collections of bioactive compounds that may possess inhibitory activity [132]. Virtual screening and molecular dynamics simulations predicted that three identified bioactive compounds (i.e., catechin, eplerenone, and oritin-4-beta-ol) are expected to bind DnaK proteins in *P. aeruginosa* and *S. aureus.* DnaK proteins are known heat shock proteins that mediate bacterial stress responses, and, thus, bioactive compounds that cripple the chaperone function of DnaK are promising drug candidates [132].

*Physicochemical characterization (PCC) of AgNPs*: Each manufacturing process gives AgNPs unique PCC properties: size distribution, agglomeration, shape, surface area, surface-to-volume ratio, chemical composition, purity, surface functionalization, surface charge, and solubility (Table 4). Numerous characterization techniques can be employed to establish these PCC properties and to predict their behaviors during application. These PCC methods are distinguished by the source of energy and the phenomenon at the origin of the signal [97]. For example, the spectroscopic characterization methods (e.g., FT-IR and Raman spectroscopy) are photon-based and may involve elastic and inelastic scattering phenomena [97]. The electron characterization methods (e.g., SEM and TEM) employ accelerated electron beams, while the thermodynamic characterization methods (e.g., TGA) use a thermodynamic parameter such as temperature or pressure as a probe [97]. 

Two of the first properties of AgNPs to be analyzed, usually by UV-Vis absorption spectrophotometry, TEM, and/or DLS, are size distribution (typically 1–100 nm) and aggregation state. These PCCs can have an impact on AgNPs’ ability to penetrate or interact with bacteria [64,138]. Smaller AgNPs can be more toxic than larger AgNPs due to their larger surface areas and larger surface-to-volume ratios (i.e., higher chemical reactivity due to a larger number of atoms present at the nanosurface) [139]. These PCCs are typically determined via Brunauer–Emmett–Teller measurements (Table 4). The chemical composition and purity of AgNPs are established by spectroscopic and microscopic techniques (e.g., Raman, ICP-OES, and SPM) to confirm the quality of AgNPs and ensure batch-to-batch reproducibility. In turn, this helps identify the PCCs that are enhancing the antibacterial activity of AgNPs. The surface charges established by the zeta potential impact the nano-stability by keeping AgNPs suspended in the colloid through electrostatic repulsions [136]. SEM and TEM showed that AgNPs can come in many shapes, such as a cube, sphere, platelet, or ring, and each will behave differently within a biological matrix [140]. Spherical AgNPs, the subject of this review and most antimicrobial studies on AgNPs, exhibit the highest antibacterial efficacy and can release significant amounts of antibacterial Ag^+^ ions. In this shape-related trend, spherical AgNPs are followed by disk-shaped AgNPs and then triangular-plate AgNPs [141]. The surface functionalization is also important because the covalent or non-covalent bonding of compounds to AgNPs leads to changes in their PCC properties and associated antibacterial mechanisms [142,143]. The characterization of the AgNP functionalization with antibiotics or non-antibiotic agents (e.g., other antibacterial agents, Raman reporters, and fluorescent tags) is even more important to verify the linkage between the two components (Figure 8) [144]. The functionalization process of AgNPs with antimicrobial agents is still in its infancy, but recent studies using intermediate ligands in between AgNPs and the antibacterial agents (e.g., DNA, RNA, amino acids, peptides, or proteins—Figure 8) show promising results [142,143,145,146]. Characterization techniques confirming the direct or indirect binding and the binding geometry of these constructs at the nanosurface include but are not limited to FT-IR, Raman spectroscopy, SERS, XPS, and NMR (Table 4). Functionalizing AgNPs can also enhance their detection capabilities with Raman reporters and fluorescent tags and reduce their toxicity by increasing the targeting capacity. For instance, glucose-stabilized silver nanoparticles (Glu-AgNPs) functionalized with a pyrimidine-based fluorescent probe have shown great ability to detect *P. aeruginosa* bacteria in water, soil, milk, cane sugar, and orange juice [146]. Targeted delivery is well established in cancer therapies, where an antibody, aptamer, peptide, or polysaccharide that is specific to a cell surface receptor is attached to the NP and employed to deliver the NP cargo to specific target cells [132]. For example, AgNPs functionalized with antimicrobial peptides and proteins using a gelatinized coating showed a fourfold or greater reduction in the MIC when compared to unfunctionalized AgNPs [145].

Overall, the PCC properties of AgNPs are interconnected, and the use of different characterization techniques for the PCC is essential in assessing their efficacy as antibacterial agents.

## 5. Antibacterial Mechanisms of AgNPs

Numerous studies report significant cell membrane and DNA damage by nearly all types of AgNPs and Ag^+^ ions, in both bacterial models [149,150,151]. Examples include but are not limited to GNB such as *P. aeruginosa* [151], *K. pneumoniae* [152], *V. vulnificus* [86], *A. baumannii* [153], *E. coli* [151], and *Enterobacter* species, and GPB such as *E. faecium* [154], *S. aureus* [155], *S. pneumoniae* [156], and *B. brevis* [157]. As illustrated below, the antibacterial mechanisms of engineered AgNPs are multifaceted and intertwined. This is because they are governed by both the different cellular structures of GNB and GPB, and the PCC properties of AgNPs (e.g., size, aggregation, surface charge, surface area, and surface-to-volume ratio) [155].

### 5.1. Cell Membrane Damage

The first, and often viewed as the most important, interaction between AgNPs and bacteria involves the plasma membrane and the components outside of this specialized structure (Figure 9). These interactions can cause physical damage through direct membrane contact, depolarization, altered permeability, osmotic collapse, leakage of K^+^ ions and other intracellular contents, and halted cellular respiration [151,155,158]. In turn, the membrane damage can facilitate additional entry of AgNPs and other cytotoxic, extracellular compounds such as antibiotics that were previously unable to pass through or were ejected by the semipermeable membrane [64].

*GNB-AgNPs:* The cell wall of a GNB cell is arranged in four layers: the outer membrane, the thin peptidoglycan layer, the periplasmic space, and the plasma membrane [160].

**The outer membrane** possesses proteins, lipids, and LPSs (lipopolysaccharides), where AgNPs and Ag^+^ ions initially interact with the bacteria [161]. For example, the negative charge of LPS in GNB has a strong attraction to positively charged AgNPs due to the large polysaccharide component [91,150]. Both GNB and GPB have an overall negative outer surface charge; the peptidoglycan components of carboxyl derivatives and phosphate groups in the GPB cell envelope are responsible for this charge [162]. Positively charged AgNPs (e.g., (NH_2_)-functionalized AgNPs synthesized with ethyleneimine) were found to exhibit a higher attraction to the bacterial cell surfaces than their negatively charged counterparts (e.g., citrate-capped AgNPs) [163]. Generally, neutral and negatively charged AgNPs have showed diminished antibacterial efficacy, with negative AgNPs being the least effective [162,164]. Nevertheless, negatively charged AgNPs can overcome this electrostatic barrier and thereby exhibit antibacterial efficacy. This was related to the formation of a protein corona around AgNPs or the charge reversal of AgNPs caused by the change in surrounding conditions [165,166,167,168]. For example, lowering the pH to acidic changed the charge of AgNPs from negative to positive [165]. This phenomenon potentially allows specific targeting of AgNP therapeutics to wound infection sites that are typically acidic. PCC properties of AgNPs such as size, surface charge, and hydrophobicity were reported to determine the type of protein corona formed around them, within a biological matrix [169]. Once formed, the protein corona improves the stability of AgNPs, promotes their cellular uptake, and generally prevents their aggregation [170,171]. Larger or unstable AgNPs that are prone to aggregation into larger AgNP clusters (≥100 nm) can exhibit reduced antimicrobial efficacy [64]. This has been demonstrated by contrasting the MIC values of citrate-capped AgNPs of 5 nm and 100 nm in *E. coli* strains (20and 110 µg mL^−1^, respectively) [64,172]. Like charge, the size of AgNPs is known to greatly influence their antibacterial activity in both GNB and GPB. It is generally accepted that AgNPs of smaller size (≤10 nm in diameter) have enhanced antibacterial activity when compared to larger AgNPs [61,172]. This was attributed to the larger nanosurface area that is available for direct contact with the bacterial cell, and the increased membrane permeability for smaller AgNPs [61,64]. Overall, the structural damage or alterations of the membrane caused by AgNPs provide a gateway for the other layers to undergo further interactions with Ag. The severity of these interactions depends on the depth and composition of the membrane layer as well as on their PCC properties [159].

**The peptidoglycan layer** is the second component of the cell envelope but makes up only a small fraction in GNB (i.e., 5–10%) [76,173]. In *E. coli,* the most widely used GNB model, the glycan strands consist of alternating *N*-acetylglucosamine (Glc*N*Ac) and *N*-acetylmuramic acid (Mur*N*Ac) residues linked by β-1→4 bonds [174]. This structure has many carboxyl groups, giving peptidoglycan a negative charge [159]. This opposition in charges results in a strong attraction to AgNPs^+^ or Ag^+^, which then adhere to the cell membrane and disrupt the cellular transport of vital molecules, the membrane potential, and the osmotic equilibrium [159,175]. It is possible that AgNPs may only attach to the outer membrane, but when AgNPs penetrate the membrane, vital intracellular processes are modified (e.g., ATP production, DNA replication, and gene expression) [159,176].

**The periplasmic space** is what divides the inner membrane from the peptidoglycan layer. Functions of the periplasmic space include cell division regulation, sequestration of enzymes that could be toxic in the cytoplasm, signaling, protein folding, protein oxidation, and protein transport [177]. There are two mechanisms present in the periplasm: catalyzation of thiol oxidation and reduction of disulfides. These pathways dispel electrons after oxidation or translocate the reducing power from the cytoplasm [178]. Thioredoxin and glutaredoxin systems play an essential role in bacteria to upkeep disulfide bonds in their reduced state in cytoplasmic proteins. AgNPs (positively or negatively charged) and Ag^+^ ions penetrating the periplasm have a very high affinity for these electron-rich groups (especially cis) and therefore, they can interrupt these enzymes and pathways [151,178,179].

**The inner membrane** is the final separation of the cytoplasm and intracellular parts from the environment in GNB. It is represented by a symmetric bilayer composed of glycerophospholipids [177]. Studies have shown that the inner membrane could be affected without damage to the outer membrane. The inner membrane is rich in ions, so leakage of these materials has been utilized to track membranolytic activity [180]. In *E. coli* and *P. aeruginosa*, depolarization of the inner membrane was noticed upon interactions with AgNPs [41]. Furthermore, K^+^ ions from the Na^+^ K^+^ ATPase pump, which helps in maintaining osmotic equilibrium and membrane potential, have been shown to leak from the inner membrane [151,175].

*GPB-AgNPs:* Like GNB, GPB plasma membranes also exhibit an overall negative charge [162]. In contrast to the GNB, the GPB models have a very thick peptidoglycan layer of 20–80 nm, which makes up approximately 90% of the cell wall [76,173]. Thus, most substances including AgNPs and Ag^+^ ions might pass with more difficulty through the peptidoglycan layers of GPB or become stuck onto the surface of the cell wall [159,181]. However, AgNPs’ contact with the peptidoglycan layer was associated with reactive oxygen species (ROS) release and the subsequent breakdown of the glycan backbone or other components (e.g., lipoteichoic acid) [182]. Furthermore, the attachment of positively charged AgNPs was found to be enhanced by the larger, negatively charged peptidoglycan [159]. Thus, positively charged AgNPs were reported to be more efficient in killing GPB than negatively charged AgNPs [162]. This is despite the lower susceptibility of GPB when compared to GNB [162].

### 5.2. Cell DNA Damage

*GNB and GPB models:* DNA damage is possible through multiple mechanisms that affect the integrity of its structure (Figure 9) [183]. Like the plasma membrane, DNA is negatively charged in both bacterial models. This is mostly due to the sugar-phosphate backbone containing a negative phosphate group (PO_4_^3−^) in each nucleotide [184]. This results in similar electrostatic attractions as also observed in the peptidoglycan layers [159]. These AgNP-DNA interactions (Figure 9) lead to DNA denaturation, DNA breaks, mutations in DNA repair genes (*mutY, mutS, mutM, mutT*, and *nth*), and interference with cell division [185]. Ag^+^ ions disrupt the double-helical structure of DNA by distorting the hydrogen bonds intercalating between base pairs [186]. Another contributor to DNA damage is the presence of ROS. AgNPs and Ag^+^ ions are foreign bodies in bacterial cells, so host-induced ROS generation will put the cell under oxidative stress and lead to apoptosis [187]. Studies of engineered AgNPs reported that smaller AgNPs have higher antibacterial efficacy and faster ROS production (e.g., 5 min for AgNPs of 1 nm in diameter versus 60 min for AgNPs of 70 nm in diameter) [188]. Under aerobic conditions, smaller AgNPs have been correlated with increased Ag^+^ ion release when compared to bulk Ag [162]. This is probably due to the larger surface-to-volume ratios characteristic of smaller AgNPs, providing a larger surface footprint for the interaction with bacteria and the subsequent Ag^+^ release [140]. For example, AgNPs of 30 nm in diameter or larger have 15–20% of its atoms on the surface, as opposed to AgNPs of 10 nm in diameter, possessing 35–40% of its atoms on the surface [189]. Overall, the oxygen radicals associated with the exposure to AgNPs and Ag^+^ ions kill pathogens through oxidative damage to amino acids, leading to DNA denaturation [190].

### 5.3. Collateral Cell Damage

*GNB and GPB models:* ROS activates other mechanisms (Figure 9) that include autophagy, neutrophil extracellular trap formation, and the triggering of pattern recognition receptors (PRRs) [190]. The oxidation of amino acids from ROS radicals consequentially results in the alteration of protein structure that jeopardizes their function. These changes can alter the protein structures, solubility, conformation, vulnerability to proteolysis, and enzymatic activity [191]. Therefore, bacterial enzymes and ribosomes are susceptible to alteration and/or denaturation as all are composed of various proteins. Bacterial ribosomes (70S) are made of ribosomal RNA and proteins, and the 70S unit is represented by a 50S and a 30S unit bonded together [192]. Ag^+^ ions are known to bind to the smaller 30S ribosomal unit that ends protein synthesis by shutting down the complex [192]. The resulting immature precursor protein buildup from AgNPs and Ag^+^ interacting with ribosomes and gene expression can lead to cell death [158].

## 6. Antibiotics against Bacteria

**Figure 10:**
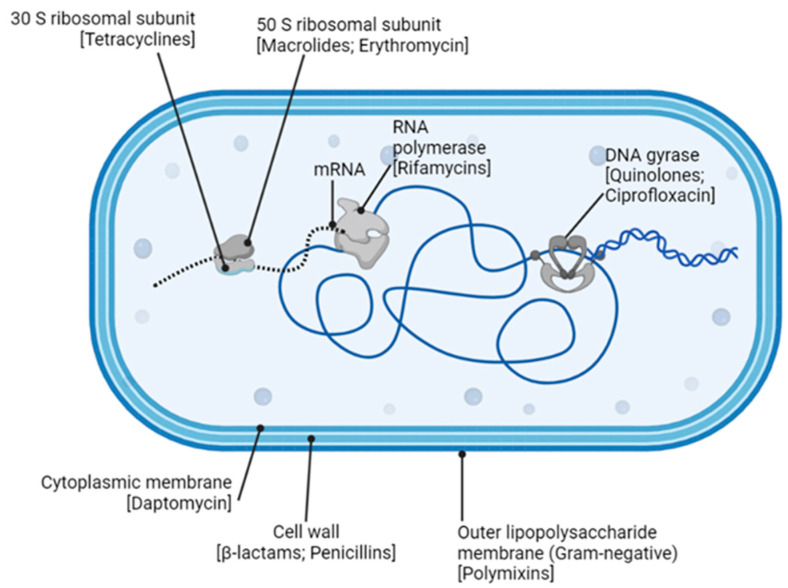
Bacterial growth targets of naturally derived antibiotics [204]. Some objects might be out of scale for illustrative purposes.

As is the case with all living organisms, bacteria compete for space, nutrients, and environments that are conducive to their existence and propagation. Antibiotic production is a natural mechanism used by microbes, including bacteria, to inhibit or kill other microbial competitors present in their environment [193]. Antibiotics are generally classified according to how they interfere with bacterial cellular growth and essential molecular processes (Figure 10) [194].

*Inhibition of cell wall synthesis:* The majority of globally produced antibiotics in use today are those that target and disrupt the bacterial cell wall. Both GPB and GNB contain layers of peptidoglycan as a constituent of the cell wall structure [195]. Each peptidoglycan layer is cross-linked to the next enveloping layer by a process called transpeptidation [195]. During bacterial growth, transpeptidases catalyze this cross-linking, resulting in a relatively strong and stable wall structure [196,197]. The β-lactam class of antibiotics (e.g., penicillin, cephalosporin, carbapenem, monobactam, and their derivatives) are so named because they all share a characteristic as part of their molecular structure—a β-lactam ring [196]. The β-lactam antibiotics bind to and inactivate the bacterial transpeptidases during new cell wall synthesis, causing loss of the cell wall entirety [198,199]. Vancomycin, a non β-lactam antibiotic, also disrupts the cell wall structure. Vancomycin belongs to a group of glycopeptide antibiotics that target the bacterial cell wall by inhibiting the synthesis of penta-peptidoglycan precursor molecules [200]. Bacitracin, a broad-spectrum cyclopeptide antibiotic, interferes with the translocation of peptidoglycan precursors across the cell membrane, so they are unable to reach, or add to, the structure of the growing cell wall [201,202,203]. In all these cases, a weaker wall results in cell death [196].

*Cell membrane disruption*: Two main groups of antibiotics can disrupt bacterial cell membranes. Polymyxins (polymyxin B and E) bind to the outer LPS membrane surface of GNB [205]. The result is an ionic imbalance across the membrane, which then becomes porous and eventually collapses [205]. This then facilitates further antibiotic entry and similar damage to the inner cytoplasmic membrane [205]. Cyclic lipopeptide antibiotics, such as daptomycin, disrupt the cytoplasmic membranes of GPB. As daptomycin binds, depolarization of the membrane occurs, resulting in a porous “leaky” barrier [206]. Membrane damage in either of these scenarios is irreparable, and so the bacterial cell dies.

*Inhibition of protein synthesis*: The aminoglycosides (streptomycin, gentamycin, kanamycin, and their derivatives) inhibit protein synthesis by interfering with bacterial ribosome function [207]. Specifically, aminoglycosides bind to the bacterial 30S (small) ribosomal subunit and cause blocking and/or misreads during translation [207]. The tetracycline group of antibiotics, such as tetracycline and doxycycline, also act via the 30S subunit, inhibiting tRNA translocation activity, and/or polypeptide chain elongation processes [204,208]. The macrolides (erythromycin, clarithromycin, and azithromycin), lincosamides (lincomycin), and streptogammin B are collectively termed the MLSB group of antibiotics [209]. Despite different molecular structures and origins, all MLSBs interact with the bacterial 50S (large) ribosomal subunit to specifically cause tRNAs, that are in the process of translocation, to prematurely drop-off the ribosome [209]. Ultimately, a drop in protein synthesis occurs that can be lethal for a growing bacterial cell [204].

*Disruption of nucleic acid synthesis and function:* Bacteria use a class of enzymes, known collectively as type II topoisomerases, to supercoil and effectively compact their DNA within the cellular space [210]. These enzymes also play an important role during DNA replication by removing supercoils upstream of the replication machinery. Once DNA synthesis is complete, the new bacterial chromosome is separated from the original and directed toward the new daughter cell. The topoisomerase that is vital for regulating the supercoiling process is DNA gyrase (topoisomerase II), while topoisomerase IV is the enzyme necessary at the end of DNA replication, since its role is to unlink newly synthesized DNA from the original [210]. The quinolone antibiotics (such as ciprofloxacin) disrupt these processes by inhibiting the DNA gyrase function in GNB and by targeting topoisomerase IV in GPB [210]. Attenuation of DNA unwinding, supercoiling, and processes imperative to its replication will ultimately lead to the cell’s demise [210].

RNA synthesis can also be interrupted by the action of antibiotics. Rifamycins (rifamycin B, SV, and derivatives) all have a characteristic macrocyclic ring structure that targets the DNA-dependent RNA polymerase enzyme (RNAP) [211]. Binding of rifamycin to the β-subunit of the RNAP stalls the transcription of DNA to RNA, which results in a significant decrease in protein production and leads to cell death [211]. 

*Disruption of metabolic pathways:* In addition to naturally derived antibiotics, a number of synthetic antibiotics have been developed to limit growth (bacteriostatic) or destroy (bactericidal) bacterial cells. Some, such as the sulfonamides and trimethoprim, are growth factor analogs that interrupt pathways involved in bacterial metabolism [212,213]. The sulfonamides are structural analogs of para-amino benzoic acid (PABA)—a vital substrate required by bacterial cells to synthesize folic acid [214]. Folic acid itself is an important vitamin used by cells to create nucleic acids. Typically, PABA forms a complex with an enzyme (dihyropteroate synthase), which then converts PABA to the folic acid precursor dihydopteric acid [214,215]. Because sulfonamide is an analog of PABA, it will also bind to this crucial enzyme, effectively outcompeting PABA and inhibiting the enzymatic production of dihydopteric acid [214,215]. Trimethoprim is a structural analog of a subsequent enzyme (dyhydrofolate reductase) in this pathway. Trimethoprim outcompetes the binding of dihydopteric acid to this secondary enzyme, thus inhibiting its activity and limiting the production of additional metabolites required for folic acid synthesis [214,215]. The synergistic effects of sulfonamides and trimethoprim cause folic acid levels to drop, which in turn inhibits nucleic acid synthesis and bacterial cell growth [214,215].

## 7. Mechanisms of Antibiotic Resistance

Unsurprisingly, antibiotic actions are countered by an array of microbial defense activities [216]. These defensive mechanisms are as ancient as the origins of prokaryotic life, steadily co-evolving with antibiotic effectiveness for the past 3.5 billion years. However, since the discovery and use of antibiotics by humankind, the rapid evolution of antimicrobial resistance has become an alarming global concern [217]. This is especially problematic in the clinical and agricultural fields, where the emergence of MDR pathogenic microorganisms is outnumbering our ability to control them effectively [218,219].

The resistant mechanisms used by bacteria are encoded in their genetic material (intrinsic resistance) or assimilated (acquired resistance) by spontaneous chromosomal mutation events, and horizontal gene transfer (HGT) (Figure 11) [216]. In a competitive environment, the expression of these genes is essential for the bacterial cell’s survival. Genes may code for enzymes that catalyze the modification or chemical breakdown of a neighboring cell’s (or their own) antibiotic, efflux pumps that move the toxic compound out of the cell, synthesis of alternative or modified antibiotic targets, and/or the use of metabolic pathways that circumnavigate antibiotic actions [216,217]. All these genes remain in the community’s genetic pool as bacterial cells divide and grow [220].

*Enzymatic inactivation:* The most well-described protective mechanism used by bacterial cells is the production of enzymes that inactivate the antibiotic by modification or destruction of its molecular structure [216,218]. For example, the β-lactamase enzymes (coded for by the bacterial gene *bla*) hydrolyze (cleave) the β-lactam ring of β-lactam antibiotics [221]. The evolution of this group of enzymes has been rapidly keeping up with the use of β-lactams and their derivatives to such an extent that more than 2000 unique β-lactamases have been described [222,223]. Bacterial synthesis of enzymes that adenylate, acetylate, and/or phosphorylate the aminoglycoside class of antibiotics (e.g., kanamycin, neomycin, and streptomycin) modify the drug’s molecular structure so effectively that its activity is inhibited, and the bacteria continue to grow [207]. The macrolides (e.g., erythromycin and azithromycin), which contain a characteristic lactone ring structure, can be degraded by hydrolyzing enzymes (esterases) produced by bacteria, or modified by phosphorylation events [224]. A current concern is the emergence of a group of destructive enzymes able to break apart the molecular structure, and inactivate the most recent next-generation synthetic tetracyclines, rifamycins, and their modified derivatives [208].

*Efflux pumps:* There are several families of efflux pumps that transport toxins, including antibiotics, out of the bacterial cell. All pumps are in the cytoplasmic membrane but can differ in their energy source (proton motive force/ATP), substrate specificity, and/or structure [225]. Regarding antibiotic resistance, the six most described efflux pump types include the major facilitator superfamily, the small-MDR-resistance family, the proteobacterial antimicrobial compound efflux family, the multidrug and toxic compound extrusion family, the ATP-binding cassette superfamily, and the resistance nodulation division family (RND) [225,226]. Many environmental bacterial strains contain more than one type of efflux pump in their membranes, and the pump itself may be able to transport a variety of antibiotics out of the cell [225,226]. Pathogenic bacteria may also possess several types of efflux pumps, in addition to overexpressing them [227]. This combination enables efficient and rapid eviction of toxic drugs from the cell’s interior. Importantly, bacteria that upregulate their efflux pump capacities include all members of the MDR ESKAPE pathogens (*E. faecium*, *S. aureus*, *K.pneumoniae*, *A. baumannii*, *P. aeruginosa*, and *Enterobacter* species) [228]. Examples from this group include *P. aeruginosa* and *A. baumannii*, which both overexpress genes associated with the RND-type efflux system to remove drugs in the quinolone, aminoglycoside, and β-lactam antibiotic classes [228]. Certainly, the use of efflux pumps by the bacterial community is a robust mechanism to counter antibiotic persistence within the cell cytoplasm [216,217,227]. 

*Target modification:* Antibiotics target a wide spectrum of vital proteins, enzymes, and metabolic pathways in a growing bacterial cell [229]. At the same time, spontaneous mutation events can result in an advantageous modification to these cellular targets so that the antibiotic is no longer effective [229]. Genes that encode alternate or modified antibiotic targets are also acquired by HGT. For example, methicillin-resistant strains of *S. aureus* (MRSA) are postulated to have received the gene *mecA*, which encodes a transpeptidase with a lowered affinity for methicillin, via HGT from a closely related ancestor [230], while chromosomal mutations in the genome of *S. pneumoniae* result in structural changes in the transpeptidases (involved in cell wall synthesis) and provide resistance to β-lactam antibiotic binding [231]. Emerging *Mycobacterium tuberculosis* (*Mtb*)-resistant strains have mutations in the DNA-dependent RNA polymerase enzyme (RNAP) that alters the structure so that it is no longer vulnerable to catalytic inhibition by the antibiotic rifampicin [232].

*Limiting antibiotic uptake and metabolic bypasses:* Porins are channels embedded in the outer LPS membrane of GNB and play a prominent role in the permeability of the membrane [233]. Translocation of toxins, such as antibiotics, can be modulated by alterations in both the number of functioning porins, and/or the use of alternative porins that have a decreased channel diameter, which effectively blocks the entry of the drug into the cell [233]. Bacterial nutritional mutants (auxotrophs) lack the metabolic capability to synthesize specific metabolites required for their survival [234,235]. If the metabolite exists in the surrounding microenvironment, the auxotroph will survive [234,235]. Because of this, antibiotics that target the enzymes involved in the synthesis of folic acid are ineffective against folic acid auxotrophs because they lack the synthesizing enzymes that the antibiotic targets [234,235].

*Horizontal Gene Transfer (HGT):* Crucially, classic vertical transfer does not limit the scope of a cell’s gene collection [236,237]. In the prokaryotic world, a major input to the cell’s arsenal of antibiotic resistance conferring genes is via their acquisition from other microbes in the surrounding community (acquired resistance) [237]. This HGT is pervasive and occurs when exogenous DNA is taken-up via three main mechanisms in nature: transformation, conjugation, and transduction [216,217,237]. Briefly, transformation describes the uptake of genetic material that is released into the environment, commonly by a lysed cell [237]. This “free” donor genetic material may enter and become part of a living cell’s genome if homologous recombination occurs successfully with the recipient’s DNA [237]. Genes conferring antibiotic resistance are not always a part of the chromosome but are often located on plasmids, the small extra-chromosomal genetic material that is common to prokaryotic cells [237]. Many of the genes are encoded on the aptly named “R” (Resistance) plasmid. During conjugation, a physical connection occurs between the donor and recipient bacterial cells. This connection facilitates the synchronized copying and transfer of plasmids (or even entire chromosomes) containing properties such as antibiotic resistance and virulence [219]. Genetic elements can also be inadvertently injected into a bacteria’s cytoplasm via a bacteriophage (bacterial virus)—a process referred to as transduction [216,217,237]. During replication of the viral particles, any fragment of the host’s genome can be accidentally assembled into the viral capsid [238]. This virus leaves the (donor) host cell to attack another bacterial host (recipient). Instead of its own genome, the virus injects genetic material from the previous bacterial host [238]. The incoming nucleic acid may share homology with the new host, and homologous recombination may follow [238]. The recipient cell has gained additional genes via a phage (the transducing particle). The selective pressure elicited by the overuse of antibiotics has resulted in the emergence of increasing numbers of resistance genes residing on mobile elements in the gene pool. As such, HGT is a major driver in the emergence of antibiotic-resistant bacteria [236].

*Biofilms and persister cells:* Biofilms are ubiquitous in nature and consist of a matrix of extracellular polymeric substances (EPSs) excreted by microorganisms [239,240]. Biofilms form on all surfaces, biotic and abiotic alike, to encompass communities of microbes and establish a niche [239,240]. The matrix itself can often limit the diffusion of antibiotics and is a major issue when treating infections caused by pathogens that are biofilm producers (Figure 12) [239]. To compound the problem, the living bacteria inside the biofilm often upregulate the production of efflux pumps and increase the secretion of enzymes into the EPS that destroy antibiotics that can navigate into the matrix [239,240]. Persister cells may also be present in the biofilm. These cells are resistant to most antibiotics since they are dormant, so they are not growing or metabolizing [239,240]. It has been proposed that these persister cells are responsible for relapses following the end of antibiotic treatment for bacterial infections [232]. Once halted, the persister cells emerge from dormancy and replicate. The biofilm becomes repopulated, and the infection returns [239,240,241].

## 8. Synergistic Effects of AgNP-Antibiotic Conjugates on Antibiotic-Resistant Bacteria

Synergy is defined as the phenomenon that combines two or more compounds, leading to a response with more potency than an individual compound can exert alone [242]. Currently, extensive efforts have been dedicated to exploiting the synergistic effects of core AgNPs functionalized with antibiotics (denoted AgNP–antibiotic conjugates) in preventing antibiotic-resistant and non-resistant pathogens from spreading or multiplying [243,244,245]. This section will focus on summarizing the advances and challenges encountered in the fabrication, characterization, and evaluation of the synergistic effects of AgNP–antibiotic conjugates on bacterial growth. Within this context, illustrative examples are presented for both antibiotic-resistant and non-resistant bacterial strains with respect to the type of AgNP–antibiotic conjugates used, and their PCC properties.

*Strategies to conjugate antibiotics to AgNPs:* Core AgNPs are fabricated chemically through the reduction of Ag^+^ from a silver salt with chemical reagents (e.g., citrate or sodium borohydride) or biogenic reagents (e.g., bacterial, fungal, or plant extracts). Antibiotics are then conjugated to AgNPs via one of the following methods: (i) conjugation of antibiotics after the AgNP synthesis or (ii) conjugation of antibiotics during the AgNP synthesis. Both methods can use the antibiotic as either a reducing agent, a functionalization agent, or both [244]. Each of these strategies have been reported to produce a wealth of AgNP–antibiotic conjugates with synergistic effects against both non-resistant and antibiotic-resistant pathogens [244]. For example, AgNP–gentamycin conjugates capped with PVP were shown to be potent antibacterial agents against *S*. *aureus*, *E. coli*, and gentamycin-resistant *E. coli* [246]. The mechanism of synergy was attributed to a multistep process: gentamycin, a neutral aminoglycoside, lowers the negative charge of AgNPs, and thereby promotes the membrane–AgNP interaction and release of Ag^+^ ions at the site of membrane attachment. Additionally, complex nanostructures such as cyanographene Ag nanohybrids (GCN/AG) conjugated with gentamicin reduced the original MIC of gentamicin by 32-fold and had an average fractional inhibitory concentration (FIC) of 0.39 [247]. Partial synergy was also found for GCN/Ag conjugated with ceftazidime against *E. coli*. Several studies address the significance of AgNP size, shape, and surface charge on synergy. These include demonstrations that positively charged AgNPs (e.g., amine-capped AgNPs) have a stronger inhibitory effect against bacteria than negatively charged AgNPs (e.g., citrate-capped AgNPs), while both AgNPs have the same nanocore (sodium borohydride reducing agent) and are conjugated with the same antibiotic (e.g., vancomycin; Van-AgNPs) [248,249]. The reported MIC values against *S aureus* were 5.7 fmol mL^−1^ for positively charged AgNPs (+50 mV), 4 nmol mL^−1^ for neutral AgNPs (0 mV), and 97 nmol mL^−1^ for negatively charged AgNPs (−38 mV). AgNP–ampicillin conjugates that were synthesized using ampicillin as a reducing agent had significantly reduced MIC values (3–28 µg mL^−1^) when compared to ampicillin (12–720 µg mL^−1^) or AgNPs alone (280–640 µg mL^−1^). Antibiotic-resistant (*E. coli* and *S. aureus*) and MDR (*P. aeruginosa* and *K. pneumonia*) bacterial strains were susceptible to ampicillin but did not develop resistance to the AgNP–ampicillin conjugates even after 15 growth cycles [250].

*Characterization of AgNP–antibiotic conjugates:* The two components (AgNPs and antibiotics) must be chemically conjugated to exhibit synergistic effects. The AgNP–antibiotic interactions (e.g., the surface functionalization) and other PCC properties are typically characterized following the U.S. EPA standards in conjunction with the techniques described in Table 4. For example, the UV-Vis absorption spectrophotometry analysis of AgNP–vancomycin conjugates exhibited a consistent red shift in the characteristic localized surface plasmon resonance (SPR) peak of the citrate-capped AgNPs (392 nm) upon binding to antibiotics [251]. This AgNP–antibiotic conjugate demonstrated synergistic antibacterial potential, rather than additive effects, against GPB (*S*. *aureus)* and GNB (*E. coli).* FT-IR measurements of citrate-capped AgNPs synthesized using *Bacillus* sp. *SJ14* showed peaks characteristic to the bending and stretching motions of primary amines at 1635 cm^−1^ and 3326 cm^−1^, respectively [252]. The spectroscopic measurements helped characterize the surface chemistry and stability of AgNPs when attached to microbial sourced proteins [252]. The subsequent conjugation of these AgNPs to antibiotics (i.e., ciprofloxacin, methicillin, and gentamicin) was confirmed through the broadening of the localized SPR absorption peaks at 420 nm, and the significant Raman shifts in the marker amine vibrational peaks (e.g., from 1635 cm^−1^ to 1652 cm^−1^) [252]. All AgNP–antibiotic conjugates showed synergy; most notably, the MIC of methicillin was reduced from 250 μg mL^−1^ to 7.8 μg mL^−1^ against an MDR-biofilm-forming coagulase-negative *S. epidermidis.* Raman spectroscopy can be utilized as a complementary tool to IR spectroscopy to characterize the change in the nanosurface chemistry upon the AgNP-antibiotic chelation. For example, the UV-Vis absorption and Raman spectra of four classes of antibiotics, *β*-lactam (ampicillin and penicillin), quinolone (enoxacin), aminoglycoside (kanamycin and neomycin), and polypeptide (tetracycline), could be collected with minimum to no sample preparation, before and after complexation with citrate-capped AgNPs [253]. Both analytical techniques confirmed the interaction between AgNPs and antibiotics, after the replacement of the citrate coating with antibiotics. All AgNP–antibiotic conjugates showed synergistic growth inhibition against MDR *Salmonella* Typhimurium DT 104, except for ampicillin and penicillin [253]. Specifically, no SERS enhancements were observed when AgNPs were combined with ampicillin and penicillin at any test concentrations (i.e., minimal to no AgNP–antibiotic interaction for these antibiotics) [253]. In contrast, distinct Raman marker bands were observed for all other antibiotics complexed to the nanosurface. For example, kanamycin was identified through vibrational modes at 270 cm^−1^ (Ag-O stretching), 620 cm^−1^, and 890 cm^−1^ (skeletal deformation and stretching of the tetrahydropyran rings) [253]. AgNPs alone reduced the bacterial growth of MDR *Salmonella* by 10%. Furthermore, tetracycline enhanced the binding of AgNPs to *Salmonella* by 21% and the Ag^+^ release by 26%, when compared to AgNPs alone. This Raman study further confirmed the relationship between the synergistic, antibacterial effects and the necessity of prior AgNP-antibiotic binding.

*Quantifying growth inhibition and synergy of AgNP–antibiotic conjugates:* Three basic procedures are typically utilized to study growth inhibition. One of the oldest methods to determine growth inhibition is the Kirby–Bauer (disk diffusion) test that maintains its popularity because it requires small volumes of sample (10–20 μL), no specialized equipment, and gives a quick turnaround [245,254]. In these assays, 6 mm filter disks are soaked with antimicrobials and placed on an agar plate coated with bacteria at 10^8^ CFU mL^−1^. Following overnight growth, the antibiotic concentration that produces a distinctive halo (mm diameter) around the disc is considered the zone of inhibition (CLSI protocol). This varies according to the antibiotic used and bacteria being tested. In the solution-based growth inhibition assay, bacterial cultures at 10^5^ CFU mL^−1^ are mixed with antimicrobials and are grown for 20–24 h [245]. The cell growth is then assessed by monitoring the optical density (OD) at 600 nm. The antibiotic concentration corresponding to OD values of ~50% below that of the untreated cells is considered the MIC. This type of assay also has a relatively fast turnaround, but it must be supplemented with colony counting to establish that the OD 600 values correspond to viable cell counts. The growth inhibition assay based on colony counting is perhaps the most labor-intense of the three methods. It is a solution-based growth inhibition, in which cells at 10^5^ CFU mL^−1^ are grown for 2 h with antimicrobials, then plated [245]. Viable colonies are then counted after 24 h of growth.

To establish a common standard for synergy, a FIC can be calculated by dividing the MIC of the AgNP–antibiotic conjugate with the MIC of antibiotic alone (Equation (1)). An FIC value of 0.5 is considered synergistic [243].

(1) $$\mathrm{FIC}=\frac{\mathrm{MIC}\;\mathrm{of}\;\mathrm{AgNP}-\mathrm{antibiotic}\;\mathrm{conjugate}}{\mathrm{MIC}\;\mathrm{of}\;\mathrm{antibiotic}\;\mathrm{alone}}$$

*Selective examples of synergy against resistant and non-resistant bacterial strains:* There is an extensive collection of evidence highlighting the potency of AgNP–antibiotic conjugates against both GPB and GNB [243,244,255,256]. Table 5 collates available data listing the method used for AgNP synthesis (biological versus chemical), the properties of AgNPs (i.e., size, charge, and shape, if available), and the antibiotic–bacteria pairs with demonstrated synergy.

*AgNP–antibiotic synergy with potential for clinical applications on MDR pathogens:* A recently published seminal study used *A. baumannii* to produce biogenic AgNPs and test their antibacterial efficacy on carbapenemase-producing Gram-negative bacteria (CPGB) alone and conjugated to various antibiotics [257]. The study found potent antimicrobial activity against CPGB, with MICs ranging from 64 to 8 μg mL^−1^. Among the conjugates, AgNP-ceftriaxone showed the highest synergistic effect with the MIC lowered by 250-fold (from 1024 μg mL^−1^ to 4 μg mL^−1^) against *A. baumannii*. This result is significant because carbapenems are considered the “last resort” option, while β-lactams are often the go-to first line of treatment against severe bacterial infections. Hence, developing new treatment options against carbapenem-resistant bacteria is imperative. *Post-surgical ointments:* Biogenic AgNPs were produced by reduction of Ag^+^ with extracts of the fungus *Fusarium oxysporum.* The potency of these AgNPs was enhanced with waxes and natural oils and used as a post-surgical ointment on goats that were infected with *Caseous lymphadenitis*. The goats receiving treatment healed faster and had fewer wound infections compared to the control group [258]. *AgNP coating prevents biofilm formation:* Biofilm formation on surgical implants is a major cause of post-surgical infections. AgNPs that were coated with polydopamine, chitosan, and hydroxyapatite on titanium implants resulted in 90–92% of antibiofilm efficiency against *S. aureus*, *S. epidermidis,* and *E coli* [259].

**Table 5:** Silver nanoparticle (AgNP)–antibiotic complexes that demonstrated synergy in reported work. The corresponding references are included.

Fabrication Method and Reducing Agent	AgNP Size and Coating	Synergy, Bacterial Model, and Method of Evaluation
Biological *Bacillus* sp. [260]	14–42 nm primary and aromatic amines	ZoI; all combinations of fusidic acid, gentamycin, ciprofloxacin, erythromycin, penicillin, chloramphenicol, levofloxacin, nalidixic acid, and ampicillin against *S. epidermidis, S. aureus, V. cholerae, S. aureus, Salmonella* Typhi, and *Salmonella* Paratyphi
Chemical [261]	10–30 nm nanosilver colloid	MIC; allicin against MRSA
Biological *Trichoderma viride* *Aspergillus flavus* [262,263]	5–40 nm	ZoI; ampicillin, kanamycin, erythromycin, and chloramphenicol against *E. coli*, *S.* Typhi, *S. aureus*, *Micrococcus luteus*, *P. aeruginosa*, *E. faecalis*, *A. baumanii*, *K. pneumoniae*, and *Bacillus* spp.
Biological *Phytophthora* *Infestans* [264]	5–30 nm	ZoI, MIC, mupirocin, neomycin, vancomycin against *S aureus;* cefazolin, mupirocin, gentamycin, vancomycin against *P. aeruginosa;* and cefazolin, mupirocin, gentamycin, neomycin, tetracycline against *E. coli*.
Chemical Maltose [265]	28 nm and 8 nm	MIC; amoxycillin, colistin, and gentamycin against *A. pleuropneumoniae,* and *P. multocida.* Penicillin G against *A. pleuropneumoniae*
Biological *Mukia* *Maderaspatana* [266]	N. A.	ZoI; biofilm microplate; cefriaxone with *B. subtilis*, *K. pneumoniae*, *S. aureus*, *S.* Typhi, and *Pseudomonas fluorescens*
Chemical ascorbic acid [267]	20 nm	MIC; amoxicillin against *E. coli*
Biological *E. hermannii, C. sedlakii*, and *P. putida* [268]	4–12 nm	ZoI; gentamicin against *P. aeruginosa* and vancomycin against *S. aureus* and MRSA
Chemical solid silver [269]	N. A.	MIC, FIC, biofilm, and hydroxyl radical assay; *E. faecium*, *S. mutans*, and *E. Coli* with ampicillin; *E. faecium* and *P. aeruginosa* with chloramphenicol; *S. aureus*, *S. mutans*, *E. coli,* and *P. aeruginosa* with kanamycin
Biological *K. pneumoniae* [270]	50 nm	ZoI; penicillin G, amoxicillin, erythromycin, clindamycin, and vancomycin against *S. aureus* and *E. coli*
Biological *Dioscorea bulbifera* [271]	8–20 nm	ZoI; chloramphenicol and vancomycin against *P. aeruginosa*, streptomycin with *E. coli*
Chemical sodium citrate and garlic [272]	- citrate-coated	ZoI; *S.* Typhi, *E. coli*, *P. aeruginosa*, *M. luteus*, *S. aureus* with amoxclav and *S.* Typhi with ampicillin
Chemical [273]	3.0 nm	FI; *E. faecium*, ampicillin and chloramphenicol; *S. mutans*, ampicillin and kanamycin; *E. coli*, ampicillin and kanamycin; *P. aeruginosa*, chloramphenicol and kanamycin
Chemical NaBH_4_/citrate [274]	5.0−12.0 nm citrate-coated	*A. baumannii* with polymyxin B and rifampicin
Chemical NaBH_4_/maltose [265]	8.0 nm gelatin-coated	FIC; *A. pleuropneumoniae*, penicillin G; *E. coli*, colistin; *S. aureus*, gentamicin
Chemical Gallic acid [275]	8.6 nm Gallic-acid-coated	FIC; *E. faecium*, *A. baumannii*, *K. pneumoniae, Morganella morganii*, and *P. aeruginosa*, ampicillin and amikacin; *S. aureus, E. coli*, and *Enterobacter cloacae*, amikacin (FIC)
Chemical NaBH_4_/citrate/hydrazine [276]	10 nm PVP- and citrate-coated	ZoI; *S. aureus*, cephalexin
Chemical NaBH_4_/citrate [277]	16 nm PVP-coated	ZoI; *E. coli*, streptomycin, ampicillin, and tetracycline; *S. aureus*, streptomycin, ampicillin, and tetracycline
Chemical NaBH_4_/citrate [278]	19.3 nm SDS-coated	ZoI; *E. coli*, streptomycin, ampicillin, and tetracycline; *S. aureus*, streptomycin, ampicillin, and tetracycline
Chemical ascorbic acid [279]	20.0 nm	MIC; *E. coli*, amoxicillin
Chemical NaBH_4_ [280]	20.0 nm PVP-coated	ZoI; all combinations of vancomycin and amikacin and *S. aureus* and *E. coli*
Chemical Tween 80 [281]	20.0–40.0 nm Tween 80-coated	FIC; *S. epidermidis* and gentamicin
Chemical citrate [246]	23.0 nm citrate-coated	MIC and inhibition (plate counting); *S.* Typhimurium, tetracycline, neomycin, and penicillin G
Chemical ethylene glycol [251]	25.0 nm PVP-coated	FIC; *E. coli* and *S. aureus*, gentamicin
Chemical Maltose [282]	26.0 nm gelatin	MIC; *E. coli*, ampicillin, ampicillin/sulbactam, aztreonam, cefazolin, cefoxitin, cefuroxime, cotrimoxazole, colistin, gentamicin, ofloxacin, oxolinic acid, and tetracycline; *P. aeruginosa*, amikacin, aztreonam, cefepime, cefoperazone, ceftazidime, ciprofloxacin, colistin, gentamicin, meropenem, ofloxacin, piperacillin, and piperacillin/tazobactam; *S. aureus*, ampicillin/sulbactam, chloramphenicol, ciprofloxacin, clindamycin, cotrimoxazole, erythromycin, gentamicin, oxacillin, penicillin, teicoplanin, tetracycline, and vancomycin
Chemical NaBH_4_/maltose [265]	28.0 nm gelatin	Amoxycillin, penicillin G, gentamicin, and colistin
Chemical Maltose [283]	28.0 nm Maltose	FIC; *E. coli* and *K. pneumoniae* with cefotaxime, ceftazidime, meropenem, ciprofloxacin, and gentamicin; synergism in all resistant strains except to *K. pneumonia* carbapenemase
Chemical Citrate [253]	29.8 nm citrate-coated	Ampicillin, penicillin, enoxacin, kanamycin, neomycin, and tetracycline, and *S.* Typhimurium
Chemical Citrate [284]	29.8 nm citrate-coated	Inhibition (plate counting); *S.* Typhimurium, enoxacin, kanamycin, neomycin, and tetracycline
Chemical NaBH_4_/citrate [271]	38.3 nm citrate-coated	ZoI; *E. coli*, streptomycin, ampicillin, and tetracycline; *S. aureus*, streptomycin, ampicillin, and tetracycline
Chemical Citrate [251]	70.0 nm Citrate-coated	ZoI; vancomycin and *S. aureus* and *E. coli*
Biological *Streptomyces cali- diresistants* IF17 strain [284]	5.0–20.0 nm biomolecules from actinobacterial strains	FIC: *E. coli*, tetracycline; *S. aureus*, ampicillin, kanamycin, and tetracycline; *B. subtilis*, ampicillin, kanamycin, and tetracycline
Biological *Klebsiella pneumoniae* extract [271]	5.0–32.0 nm proteins from biomass	ZoI; *E. coli*, amoxicillin, erythromycin, penicillin, and vancomycin; *S. aureus*, amoxicillin, erythromycin, penicillin, and vancomycin
Biological *Streptomyces calidiresistants* IF11 [284]	5.0–50.0 nm biomolecules from reducing strains	FIC; *B. subtilis*, kanamycin
Biological *Actinomycetes* strains [277]	17.0 nm proteins from biomass	MIC and ZoI; *E. coli*, *K. pneumoniae*, and *P. aeruginosa,* ampicillin
Biological *Klebsiella pneumoniae* [285]	20.0 nm -	ZoI; *E. faecalis*, chloramphenicol and gentamicin
Biological silver-resistant estuarine *P. aeruginosa* strain [286]	35.0–60.0 nm biomolecules from reducing strains	ZoI; all combinations of ampicillin and ciprofloxacin with resistant *S. aureus* strain VN3 and ciprofloxacin-resistant *V. cholera* strain VN1
Biological *Trichoderma viride* [287]	5.0–40.0 nm proteins from biomass	ZoI; *E. coli*, ampicillin, chloramphenicol, erythromycin, and kanamycin; *M. luteus*, ampicillin, chloramphenicol, and kanamycin; *S.* Typhi, ampicillin, chloramphenicol, erythromycin, and kanamycin; *S. aureus*, ampicillin, chloramphenicol, erythromycin, and kanamycin
Biological *Acinetobacter calcoaceticus* [288]	8.0–12.0 nm biomolecules from reducing strains	ZoI or MIC; *A. baumannii*, amikacin, amoxicillin, ampicillin, chloramphenicol, ciprofloxacin, doxycycline, gentamicin, tetracycline, trimethoprim, and vancomycin; *Klebsiella* (previously known as *Enterobacter*) *aerogenes*, amikacin, amoxicillin, ampicillin, ceftriaxone, chloramphenicol, ciprofloxacin, doxycycline, gentamicin, kanamycin, penicillin, tetracycline, trimethoprim, and vancomycin; *E. coli*, amikacin, amoxicillin, ampicillin, ceftazidime, ceftriaxone, chloramphenicol, ciprofloxacin, doxycycline, gentamicin, kanamycin, penicillin, tetracycline, trimethoprim, and vancomycin; *P. aeruginosa*, amikacin, amoxicillin, ampicillin, ceftazidime, ceftriaxone, chloramphenicol, ciprofloxacin, doxycycline, gentamicin, kanamycin, penicillin, tetracycline, trimethoprim, and vancomycin; *S.* Typhimurium, amikacin, ampicillin, ceftazidime, ceftriaxone, chloramphenicol, ciprofloxacin, doxycycline, gentamicin, kanamycin, penicillin, tetracycline, trimethoprim, and vancomycin; *Shigella sonnei*, amikacin, amoxicillin, ampicillin, ceftazidime, ceftriaxone, ciprofloxacin, chloramphenicol, ciprofloxacin, doxycycline, gentamicin, kanamycin, tetracycline, trimethoprim, and vancomycin; *S. aureus*, amikacin, amoxicillin, ampicillin, ceftazidime, ceftriaxone, chloramphenicol, ciprofloxacin, doxycycline, gentamicin, kanamycin, penicillin, tetracycline, trimethoprim, and vancomycin; *S. mutans*, amikacin, amoxicillin, ampicillin, ceftazidime, ceftriaxone, chloramphenicol, ciprofloxacin, doxycycline, kanamycin, penicillin, tetracycline, trimethoprim, and vancomycin
Biological *Cryphonectria* sp. [289]	30–70 nm	ZoI; *S. aureus*, *S.* Typhi, and *E. coli*, streptomycin
Biological *Emericella nidulans* [290]	66.7 nm biomolecules from biomass	FIC; *E. coli*, amikacin and streptomycin
Biological *Aspergillus flavus* [290]	81.1 nm biomolecules from biomass	FIC; *E. coli*, amikacin and streptomycin; *S. aureus*, kanamycin, oxytetracycline, and streptomycin
Biological *Dioscorea bulbfera* [272]	2.0 nm biomolecules from biomass	ZoI; all combinations of treptomycin, rifampicin, chloramphenicol, novobiocin, and ampicillin in *E. coli*, *P. aeruginosa*, and *S. aureus*
Biological *Dioscorea bulbifera* [291]	5.0–30.0 nm proteins from biomass	ZoI; *A. baumannii*, amoxicillin, ampicillin, cefotaxime, erythromycin, gentamycin, kanamycin, nalidixic acid, nitrofurantoin, penicillin, piperacillin, rifampicin, and rimethoprim; *B. subtilis*, ampicillin, cefotaxime, chloramphenicol, nalidixic acid, nitrofurantoin, penicillin, piperacillin, streptomycin, trimethoprim, and vancomycin; *E. cloacae*, amikacin, amoxicillin, erythromycin, nalidixic acid, and penicillin; *E. coli*, amikacin, erythromycin, kanamycin, nalidixic acid, polymyxin, streptomycin, and trimethoprim; *Haemophilus influenzae*, cefotaxime, ceftriaxone, nitrofurantoin, and trimethoprim; *K. pneumoniae*, amoxicillin, ampicillin, chloramphenicol, erythromycin, feropenem, nitrofurantoin, penicillin, rifampicin, trimethoprim, and vancomycin; *Neisseria mucosa*, amikacin, ampicillin, erythromycin, feropenem, gentamycin, nitrofurantoin, penicillin, polymyxin, tetracycline, trimethoprim, and vancomycin; *Proteus mirabilis*, erythromycin, nalidixic acid, and vancomycin; *P. aeruginosa*, amikacin, amoxicillin, ampicillin, chloramphenicol, doxycycline, erythromycin, feropenem, gentamycin, kanamycin, nalidixic acid, nitrofurantoin, penicillin, streptomycin, trimethoprim, and vancomycin; *S.* Typhi, amikacin, amoxicillin, ampicillin, cefotaxime, ceftriaxone, chloramphenicol, erythromycin, gentamycin, kanamycin, nalidixic acid, nitrofurantoin, penicillin, piperacillin, polymyxin, streptomycin, trimethoprim, and vancomycin; *Serratia odorifera*, ceftazidme, erythromycin, nalidixic acid, nitrofurantoin, trimethoprim, and vancomycin; *S. aureus*, amikacin, amoxicillin, ampicillin, ceftazidme, erythromycin, kanamycin, nalidixic acid, polymyxin, streptomycin, and trimethoprim; *Vibrio parahemolyticus*, ampicillin, cefotaxime, ceftriaxone, kanamycin, nalidixic acid, nitrofurantoin, polymyxin, and trimethoprim
Biological *Argyreia nervosa* [292]	5.0–40.0 nm biomolecules from biomass	ZoI; *S. aureus*, amoxicillin/clavulamic acid, ciprofloxacin, erythromycin, gentamicin, streptomycin, tetracycline, and vancomycin; *E. coli*, amoxicillin/clavulamic acid, erythromycin, streptomycin, tetracycline, and vancomycin
Biological *Gum kondagogu* [293]	5.8 nm biomolecules from biomass	FIC; *S. aureus*, gentamicin and streptomicin; *S. aureus*, streptomicin; *E. coli*, streptomicin; *P. aeruginosa*, streptomicin
Biological *Rosa damascenes* [294]	7.4–18.3 nm	ZoI; cefotaxime with *E. coli* and MRSA
Biological *Ulva fasciata* [295]	15.0 nm	ZoI; *E. coli*, cefotaxime, cefuroxime, fosfomycin, chloramphenicol, azithromycin, and gentamicin; *Salmonella enterica*, azithromycin, gentamicin, oxacillin, cefotaxime, neomycin, ampicillin/sulbactam, cefuroxime, fosfomycin, chloramphenicol, and oxytetracycline; *S. aureus*, azithromycin, oxacillin, cefotaxime, neomycin, ampicillin/sulbactam, cefuroxime, fosfomycin, chloramphenicol, and oxytetracycline
Biological *Eurotium cristatum* [296]	15.0–20.0 nm biomolecules from biomass	ZoI; all combinations of vancomycin, oleandomycin, ceftazidime, rifampicin, penicillin G, neomycin, cephazolin, novobiocin, carbenicillin, lincomycin, tetracycline, and erythromycin, and *Candida albicans, P. aeruginosa*, and *E. coli*
Biological *Urtica dioica Linn.* [297]	20.0–30.0 nm biomolecules from biomass	ZoI; *B. cereus*, streptomycin, amikacin, kanamycin, vancomycin, tetracycline, ampicillin, cefepime, amoxicillin, and cefotaxime; *S. epidermidis*, streptomycin, amikacin, kanamycin, tetracycline, ampicillin, cefepime, and amoxicillin; *S. aureus*, streptomycin, amikacin, kanamycin, vancomycin, tetracycline, cefepime, amoxicillin, and cefotaxime; *B. subtilis*, streptomycin, amikacin, kanamycin, vancomycin, tetracycline, ampicillin, cefepime, amoxicillin, and cefotaxime; *E. coli*, streptomycin, amikacin, vancomycin, tetracycline, ampicillin, cefepime, amoxicillin, and cefotaxime; *S.* Typhimurium, streptomycin, amikacin, kanamycin, vancomycin, tetracycline, ampicillin, cefepime, amoxicillin, and cefotaxime; *K. pneumoniae*, streptomycin, amikacin, kanamycin, vancomycin, tetracycline, ampicillin, cefepime, amoxicillin, and cefotaxime; *Serratia marcescens*, streptomycin, kanamycin, tetracycline, ampicillin, amoxicillin, and cefotaxime
Biological *Zea may* [298]	45.3 nm biomolecules from biomass	ZoI; *B. cereus, E. coli*, *Listeria. monocytogenes, Salmonella* Typhimurium, and *S. aureus*, kanamycin and rifampicin
Commercial N. A. [299]	10.0–15.0 nm N. A.	Optical density; ampicillin, kanamycin, gentamycin, and clindamycin with *A. baumannii*
Commercial N. A. [300]	15.2 nm starch	FIC; *Burkholderia pseudomallei* with meropenem and gentamicin sulfate
Commercial N. A. [301]	35.0 nm PVP	FIC; *E. coli*, *Salmonella* Typhimurium, and *S. aureus,* kanamycin

Abbreviations: NA—not applicable; FIC—fractional inhibitory concentration; MIC—minimum inhibitory concentration; and ZoI—zone of inhibition.

*Overcoming bacterial antibiotic resistance using AgNP–antibiotic conjugates:* AgNP-antibiotic conjugates are less likely to promote the emergence of bacterial antibiotic resistance because they use a multifaceted approach to attack bacteria. In contrast, chemical antibiotics target a specific process in the bacterial cell such as protein synthesis or DNA replication, making it easier for the bacteria to counter their action. Figure 13 summarizes how AgNP–antibiotic conjugates evade bacterial defenses.

The inherent antibacterial properties of AgNPs promote antibiotic function by overcoming various drug resistance mechanisms.

*AgNPs trigger the production of ROS that destabilize the bacterial membrane, allowing entry of antibiotics*: AgNPs alone are potent weapons against MDR-resistant bacteria. AgNPs synthesized with extracts of *Areca catechu* exhibited potency against vancomycin-resistant *E. faecalis* (MIC of 11.25 µg mL^−1^) and MDR *A. baumannii* (MIC of 5.6 µg mL^−1^) [301]. AgNPs fabricated with *Convolvulus fruticosus* extracts were effective against MDR *E. coli* (17.1 µg mL^−1^), *K. pneumoniae* (4 µg mL^−1^), and *P. aeruginosa* (2 µg mL^−1^). Studies indicated that biogenic AgNPs accumulate at the surface of bacteria, leading to the concomitant release of Ag^+^ ions and production of ROS. AgNPs facilitate ROS release by competing with amino acids (especially *cis*) that coordinate Fe-S clusters in complexes of the electron transport chain [302]. While some ROS may be eliminated by bacteria, large amounts of ROS destabilize the bacterial cell membrane due to lipid peroxidation [303]. As a result, antibiotics that would otherwise be prevented from entering the cell are allowed to seep through the damaged membrane. Likewise, antibiotics that interfere with cell wall synthesis can provide an entryway for AgNPs. Ampicillin–AgNP synergy was found to be based on this mechanism, where ampicillin interfered with cell wall synthesis, facilitating a porous entryway for AgNPs [264].

*AgNPs interfere with the action of efflux pumps*: The efflux pump mechanism is one of the most common defenses against tetracycline antibiotics. Gold NPs were observed to downregulate efflux pump production using EtBr (a common efflux pump substrate) as well as the production of other membrane proteins that play a crucial role in membrane stability [304].

*Evading enzymes that destroy antibiotics*: One of the best-known resistance mechanisms is the enzymatic destruction of antibiotics through the action of β-lactamase (against ampicillin) and chloramphenicol acyltransferase. In this scenario, AgNPs carrying antibiotics act as a Trojan-horse delivery vehicle camouflaging their cargo [303]. *Treatment of intracellular bacteria by antibiotic conjugates:* Intracellular bacteria are species that produce infection by residing in mammalian cells, as is seen with the bacterial pathogen *M. tuberculosis*. Treatment of these conditions is challenging because most antimicrobials cannot easily enter mammalian cells [302]. In contrast, the mechanism used by mammalian cells to internalize NPs via receptor-mediated endocytosis is well established [302]. AgNPs chelated by antibiotics may provide an avenue for treating ailments caused by intracellular bacteria. One example is Ag/ZnNPs encapsulated in poly(lactic-co-glycolic acid) that delivered rifamycin to mammalian cells infected with *M. tuberculosis* [305]. These AgNP–antibiotic conjugates can employ multiple mechanisms to combat bacterial infections, making it difficult for bacteria to develop effective resistance.

*Challenges and future directions*: Even though AgNP–antibiotic conjugates have demonstrated potential in treating infections caused by MDR bacteria, there are few U.S. FDA-approved treatments that use AgNP–antibiotic conjugates [303]. To address this, improvements must be made in batch-to-batch reproducibility of AgNP during synthesis (especially biological), developing dose–response relationships, and advancing the use of organism models (in vitro and in vivo) to bridge the gap between promising potential and the establishment of AgNP–antibiotic conjugates as effective treatments. Specific targeting is expected to reduce dosage and limit toxicity: AgNP–drug complexes that do not specifically target their cargo can interact with non-target cells, while targeting strategies can lead to enhanced therapeutic effects. Two common strategies for targeting AgNP-antibiotic cargo are antibody and aptamer targeting. In the antibody method, AgNPs are covalently labeled with antibodies (e.g., AuNPs labeled with *S. aureus* antibodies) to build a construct that can selectively kill bacteria [305]. In the aptamer strategy, antibodies made of 20–80 nucleotide ssDNA or RNA (i.e., aptamers) are first developed in vitro using the systematic evolution of ligands by exponential enrichment (SELEX) to target specific ligands such as small molecules, membrane proteins, peptidoglycans, and whole cells. The aptamers are then chemically attached at one end to the AgNPs through covalent bonds. The advantages of aptamers over protein antibodies are numerous and include the ease of chemical attachment to AgNPs, potential labeling with fluorophores for target detection, higher thermal stability, and bypassing of the immune system. For example, conjugated gold nanorods have been successfully targeted against MRSA’s surface [306]. Furthermore, silver nanoclusters (AgNC) bridged by DNA aptamers specific to *S. aureus* targeted its cargo six-times more effectively compared to AgNC containing scramble (non-specific) DNA sequences [306]. Overall, specific targeting of pathogens can reduce the therapeutic dosage of antibiotics and AgNPs.

## 9. Toxicity of AgNPs

**Figure 1:**
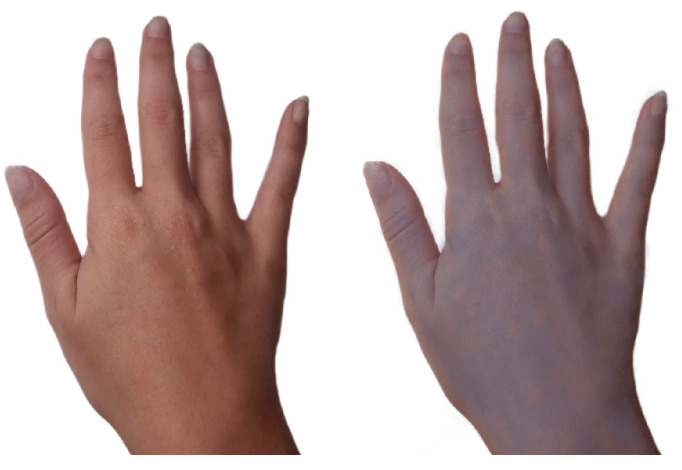
Comparison of an argyria-like skin color characteristic to topical cyanosis (**right**), versus a simulated standard hand coloring in a healthy patient (**left**).

**Figure 2:**
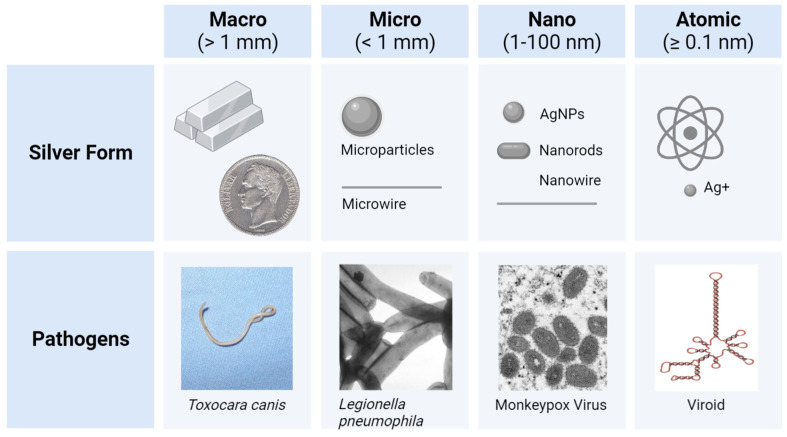
The forms of silver (Ag) utilized from B.C.E until present day, having sizes ranging from visible to the naked eye (1 mm and above) to approximately 0.1 nm (atomic radius and above) [35,37,38,39,40]. Various pathogens within each category (e.g., the *Toxocara canis* [usually 2–10 cm] and the pinworm *Enterobius vermicularis* [usually 1–3 mm] for macro pathogens >> 1 mm) depict a size comparison for the Ag forms [41,42,43,44]. Comparative scales are approximated.

**Figure 4:**
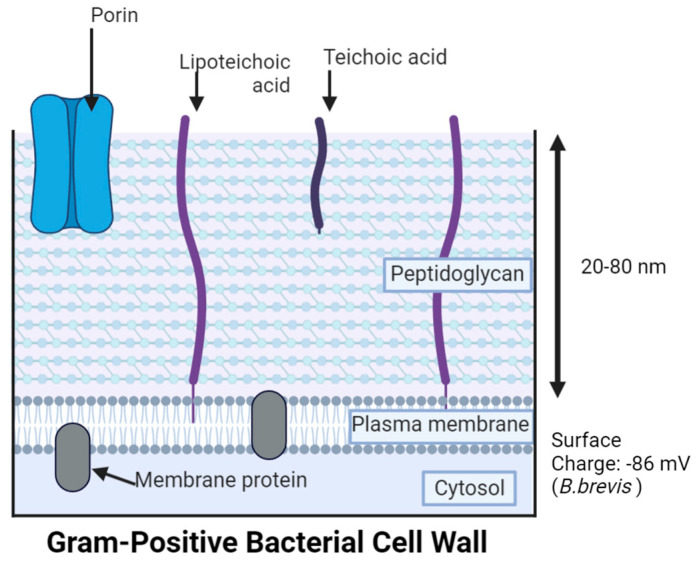
Gram-positive bacteria (GPB) wall with a thickened peptidoglycan layer, lipoteichoic acids, and teichoic acids that are exclusively characteristic to GPB. Some objects might be out of scale for illustrative purposes.

**Figure 5:**
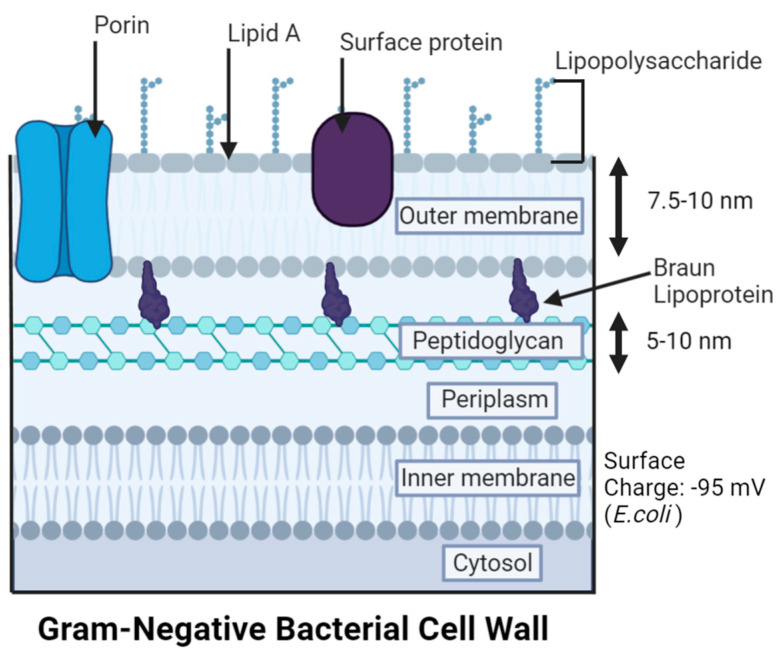
Gram-negative bacteria (GNB) wall showing its unique components: A thin peptidoglycan layer, lipopolysaccharide (LPS), and lipid A [82]. Some objects might be out of scale for illustrative purposes.

**Figure 6:**
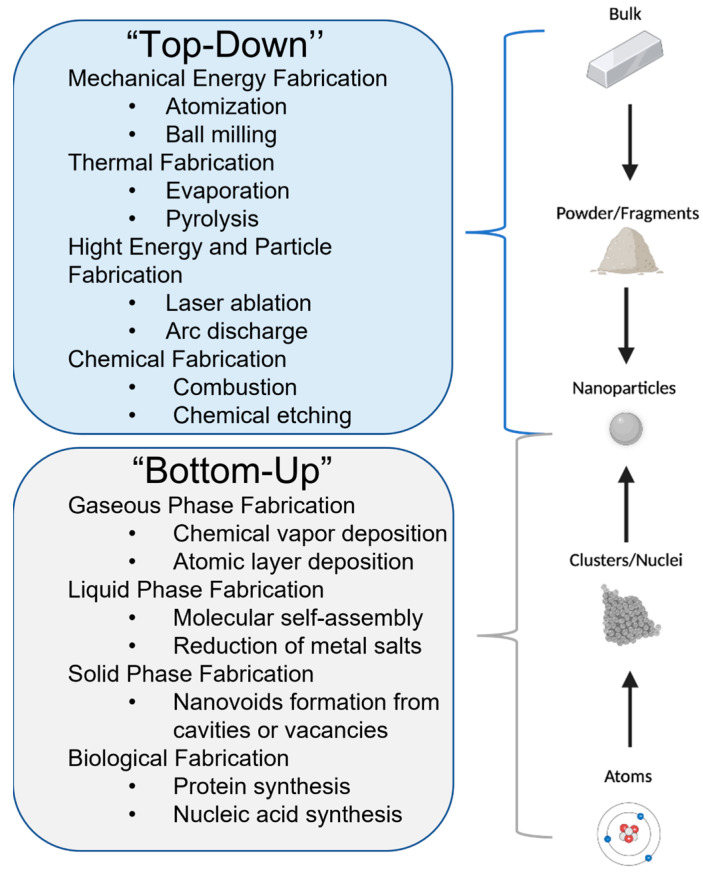
A schematic showing the formation of silver nanoparticles (AgNPs) through “top-down” versus “bottom-up” methods. A few illustrative approaches [97,98,99,100,101,102,103,104,105,106,107,108,109,110,111,112,113] are listed for each category.

**Figure 7:**
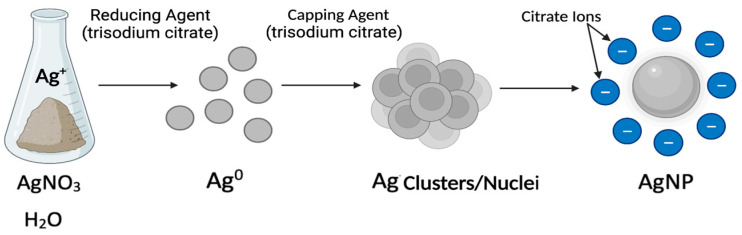
Schematics of the chemical synthesis process of colloidal, citrate-capped silver nanoparticles through the reduction of Ag^+^ ions from AgNO_3_ with trisodium citrate [116,123,124]. Some objects might be out of scale for illustrative purposes.

**Figure 8:**
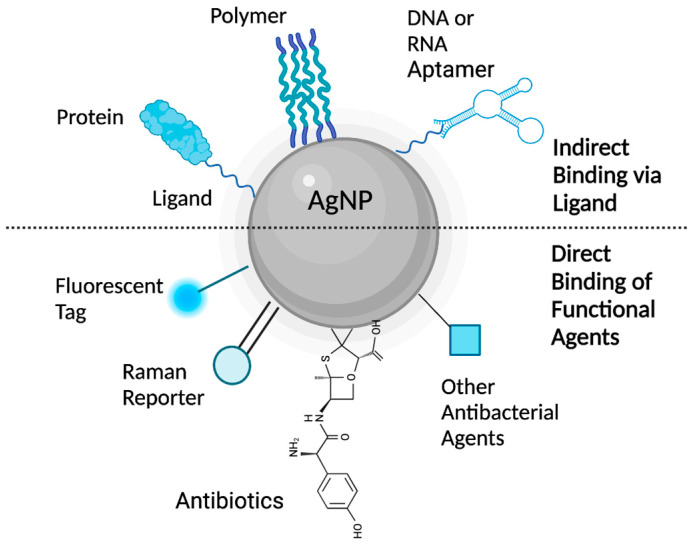
Potential direct and indirect (via ligands) binding of functional agents to the nanosurface for enhancing the antibacterial activity, the specificity of delivery, and the imaging capabilities of silver nanoparticles (AgNPs) [136,142,143,145,146,147,148].

While all bacteria are classified as prokaryotes, eukaryotes such as animals, plants, fungi, and humans are made up of cells that possess a membrane-bound nucleus containing genetic material and membrane-bound organelles [307]. Although AgNPs are recognized as efficient antibacterial agents, they can also be toxic to eukaryotic cells and larger organism models. The toxicity of AgNPs in eukaryotes remains a topic of debate due to the high complexity of multicellular organisms and the large variations among the PCC properties of AgNPs. Although a significant amount of toxicity work has been completed for both in vitro and in vivo eukaryotic models, no U.S. EPA National Primary Drinking Water Guidelines have been established yet for AgNPs [308]. National Primary Drinking Water Regulations are primary standards and treatment techniques for public water systems that are enforceable by law for the protection of public health. National Secondary Drinking Water Guidelines were determined for potential contaminants such as Ag^+^ ions, which are not considered a human health risk and are only tested for aesthetic considerations (e.g., taste, odor, and color), on a voluntary basis [309]. Human consumption of water containing Ag^+^ ions in amounts higher than the secondary maximum contaminant level (SMCL) of 0.1 mg L^−1^ was found to cause skin discoloration and graying of the white part of the eye [309]. According to the WHO, a dose of 10 g of silver nitrate (AgNO_3_) containing Ag^+^ ions can be lethal to humans, but 0.6–0.9 g of Ag^+^ may only cause argyria (Figure 1) [310]. With the low degree of regulation regarding AgNPs, further research is necessary to determine the safe levels of AgNP exposure through consumer products, medical treatments, involuntary ingestion (water and food), or work-related inhalation. To address this, numerous in vitro, in vivo, and human-related studies are being conducted to define the toxicity of AgNPs.

### 9.1. In Vitro Studies

**Figure 9:**
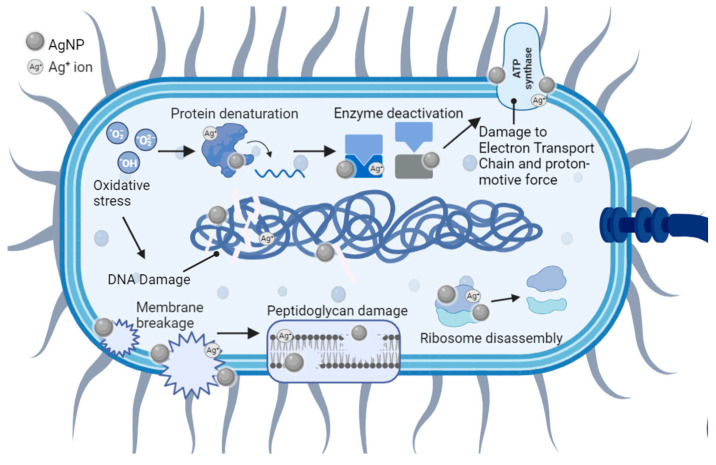
Antibacterial mechanisms of AgNPs at the membrane and the intracellular level. Some objects might be out of scale for illustrative purposes [159].

**Figure 11:**
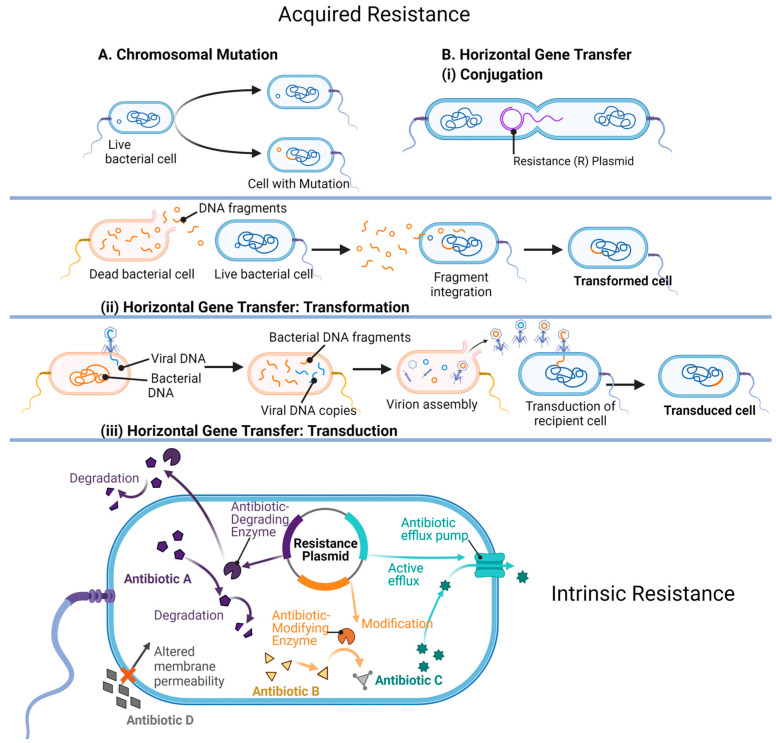
Mechanisms of antibiotic resistance in bacteria [218,220,221]. Some objects might be out of scale for illustrative purposes.

**Figure 12:**
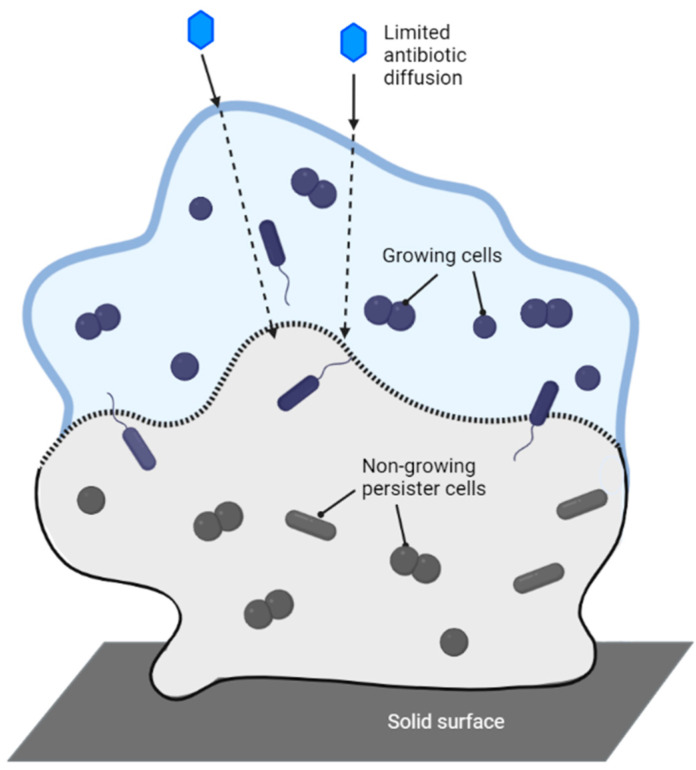
Microbial biofilm formation can limit the diffusion of antibiotics (dotted lines represent possible limit of trajectory). Some objects might be out of scale for illustrative purposes.

**Figure 13:**
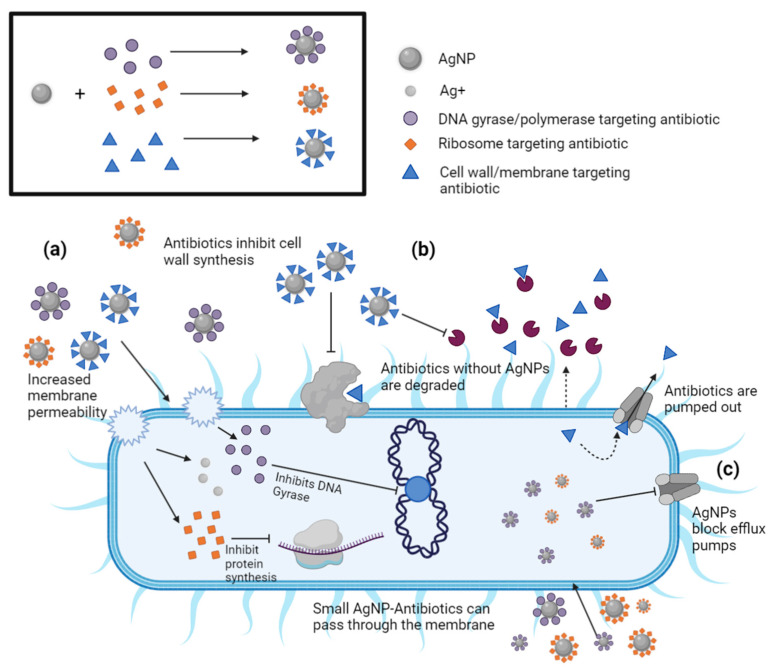
Schematic of the multifaceted antibacterial mechanisms of action of AgNP–antibiotic complexes: (**a**) AgNPs destabilize the cell wall permitting antibiotic entry through production of ROS, (**b**) antibiotics attached to AgNPs are camouflaged from the action of antibiotic-destroying enzymes, and (**c**) efflux pumps are downregulated or blocked by AgNPs. Some objects might be out of scale for illustrative purposes.

**Figure 14:**
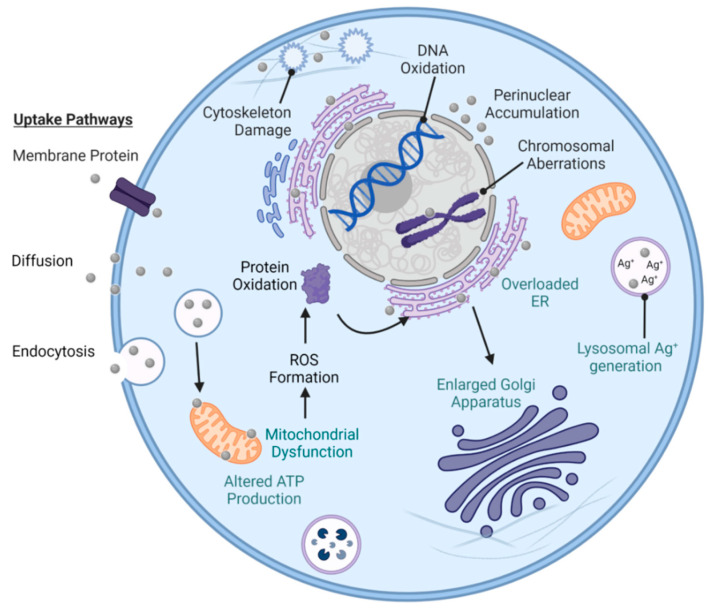
Damage mechanisms of AgNPs in eukaryotic cells. The teal text represents damage exclusive to eukaryotes, while the black text refers to damage characteristic to both bacteria (prokaryotes) and eukaryotes. Imagery and figure conceptualization were derived from [162,281,312]. Some objects might be out of scale for illustrative purposes.

The in vitro toxicity of AgNPs is influenced by the cell type, the PCC properties of AgNPs, and the exposure conditions (e.g., pH, concentration, and duration) [53]. The difficulty in addressing the cytotoxicity of AgNPs lies in the large variety of eukaryotic cells and the unique way each cell type absorbs, distributes, metabolizes, and excretes AgNPs. Pathways of AgNP-mediated damage found in bacteria (e.g., ROS formation and DNA damage—Figure 9) are shared with eukaryotic cells, but the additional organelles and differing structural organization require further understanding. Overall, these key damage mechanisms (Figure 14) have resulted in cell cycle arrest and ceased proliferation in eukaryotes [311]. Additional damage mechanisms exclusive to eukaryotes originate from the organelle uptake of intracellular AgNPs. The *mitochondrion* is one of the most researched organelles with respect to AgNP–organelle interactions because of its key role in cellular energy production and various cellular activities. AgNP–mitochondrial contact induces dysregulation of ATP production, ROS formation, and mitochondrial-mediated apoptosis [312,313]. For example, exposure of PC-12 cells, a variety of rodent neuronal cell, to 10 µg mL^−1^ of spherical AgNPs (57.2 ± 21.6 nm), for 6 h, caused mitochondrial structural changes and subsequent disruption of mitochondrial function [314]. Intracellular ROS then interfered with the function of the *endoplasmic reticulum (ER)*, which has been correlated with the accumulation of AgNPs. This has been utilized as a potential biomarker for in vitro and in vivo nanotoxicity studies [315]. For instance, multiple forms of ER stress were observed after 18 h of exposure of human retinal pigment epithelium cells to 5 µg mL^−1^ of spherical AgNPs (6.3 ± 0.62 nm) [316]. In another study, the secretory pathway of Wistar rat neuronal cells was found to be overloaded with additional protein secretion from the ER after 21 days of exposure to spherical AgNPs (10 ± 4 nm) [317]. This cellular attempt of excreting AgNPs led to the enlargement of the *Golgi apparatus* in response to the ER stress [317]. *Lysosomes* serve as another potential destination for AgNPs upon cellular entry. The AgNP–lysosome interactions modified the constitution of the lysosome including the intra-lysosomal pH and the structural integrity of the lysosomal membrane [318,319]. The biochemical degradation of AgNPs within the acidic environment of lysosomes can induce significant releases of Ag^+^ in the eukaryotic cell and cause high oxidative stress [53,319]. Such changes were reported for the endolysosomal environment of neural PC-12 cells after 1 h of contact with 10 µg mL^−1^ of spherical AgNPs (57 ± 21 nm) [314]. In another study, the highest accumulation of spherical AgNP (50 ± 20 nm, PVP-coated) was observed within lysosomal structures rather than the nucleus, Golgi complex, or ER of human mesenchymal cells. However, AgNPs were agglomerated around the eukaryotic nucleus at concentrations of 20 µg mL^−1^ or higher [320]. Altogether, these in vitro studies allow for a better understanding of the toxicity of AgNPs in specific cell lines and within controlled physical and chemical environments. The in vitro results can then be extrapolated to in vivo models of higher relevance and reliability, dealing with an internal, more complex environment of a living organism.

### 9.2. In Vivo Studies

Most in vivo toxicity studies of AgNPs addressed their interactions with different structures of a living organism under physiological conditions. AgNPs were found to be toxic to the skin, liver, lung, brain, vascular system, and reproductive structures of animal test subjects [321,322]. The breaching of other important biological barriers has also been a long-time concern and a subject of animal testing with AgNPs. For example, AgNPs were observed to cross the blood–testis barrier (10 nm, citrate-coated) [323], the placental barrier (18–20 nm, spherical) [324], and the blood–brain barrier (49.7 ± 10.5 nm, spherical, citrate-coated) in mice [325]. Significant efforts continue to be dedicated to identifying nontoxic AgNP concentrations and the organs with the most AgNP accumulation after exposure. In these studies, the most common techniques of AgNP administration to animal subjects (i.e., inhalation, oral dosages, and dermal absorption) resemble those found in humans.

*Injection and inhalation:* One such study using male and female Wistar rats established that spherical AgNPs (13–35 nm, dispersed in ethylene glycol), which were injected intravenously, induced cellular stress responses with later recovery at doses ≥10 µg mL^−1^, and exhibited accumulation in organ tissues at doses ≥20 µg mL^−1^ of AgNPs [326]. Data from another study of 90-day inhalation (low dose: 49 µg m^−3^, medium dose: 133 µg m^−3^, and high dose: 515 µg m^−3^) in male and female Sprague Dawley rats indicated that the lungs and liver accumulated the largest amounts of spherical AgNPs (18–19 nm). In the same study, oral ingestion of AgNPs had a low impact on the respiratory tract. AgNPs were also detected in the brain, yet the area with the most build-up was recorded in the olfactory bulb [327]. Another report on 10 days of inhalation (4 h daily) of 3.3 mg m^−3^ of AgNPs (5 ± 2 nm, PVP-coated) revealed low pulmonary inflammation in male C57BL/6 mice and a threefold reduction in the amount of lung accumulation over time. This decrease was from 31 µg g^−1^ of AgNPs per dry weight immediately after the final exposure down to 10 µg g^−1^ of AgNPs at three weeks after the final exposure [328].

*Oral ingestion:* Oral intake of AgNPs is another widely used technique for AgNP exposure testing on animals [329,330,331]. For example, 28 days of ingestion through oral gavage of 12.6 mg of AgNPs kg^−1^ of body weight (14 ± 4 nm, spherical, PVP-coated) led to the following accumulation levels in female Wistar Hannover Galas rats: 27 µg g^−1^ in the small intestine, 5 µg g^−1^ in the stomach, 2.5 µg g^−1^ in the kidneys, and 1 µg g^−1^ in the liver [329]. In contrast, male CD-1(ICR) mice (10 nm, spherical, citrate-coated) exhibited the highest AgNP accumulation in the brain, followed by the testis, liver, and spleen after 4 weeks of oral gavage [331].

*Dermal absorption:* Dermal penetration has been observed in both animal and human subjects with varying results of both considerable and negligible effects [332]. For example, the pig’s skin was reported to have higher permeability to spherical AgNPs (20 nm, PEG, citrate, and branched polyethyleneimine (bPEI) coatings) than human cadaver skin [333]. For the citrate-coated AgNPs, these levels were 14.02 ± 8.01 µg of AgNPs g^−1^ of skin in pigs versus 3.14 ± 1.97 µg of AgNPs g^−1^ of skin in humans [333]. However, the majority of AgNPs were left unabsorbed by both the human and pig skin and the surface charge of AgNPs (positive versus negative) did not appear to enhance penetration in human skin. In contrast, negatively charged, citrate- or PEG-capped AgNPs had a slightly higher permeability than positively charged, bPEI-capped AgNPs in pig skin [333]. In another dermal study on male and female adult zebrafish (*Danio rerio*), 24 h of exposure to 30 and 120 mg L^−1^ of spherical AgNPs (5–20 nm) contributed to ROS formation and DNA damage to hepatocytes, and enhanced expression of p53 as a response to DNA damage leading to apoptosis and subsequent necrotic sites [334]. The liver has been another principal organ of testing because of its key detoxification role. In the same zebra fish study, the metallothioneins—heavy-metal-complexing proteins within the liver—also experienced an enhanced dose-dependent expression after exposure to 120 µg mL^−1^ of AgNPs, i.e., up to 7.1-fold higher levels than the basal level for excretion [334]. The 24 h median lethal concentration (LC_50_) was established at 250 mg Ag L^−1^ for the zebra fish, and the silver buildup within the harvested liver tissues was 0.29 ng mg^−1^ for 30 mg L^−1^ of AgNP exposure and 2.4 nm mg^−1^ for 120 mg L^−1^ of AgNP exposure [334]. Overall, these in vivo studies on animal models can serve as a reference point to human studies.

### 9.3. Human Studies

There are very few reports on the short- and long-term effects of AgNP exposure in human subjects. These studies are mostly related to antibacterial applications of AgNPs [335].

*Excretion of AgNPs:* Ag present within a human body can be expelled in-part over time due to the multiple excretion pathways (e.g., biliary fecal, urinary, hair, and nail growth) [336]. AgNPs can still experience chemical interactions and transformations with cells within the human body before excretion. Ag metabolism is controlled by the induction and binding to metallothionein proteins, which protect cells and tissues against toxicity from heavy metals [336]. If Ag is ingested, it can enter the blood circulatory system as a protein complex and can be later excreted by the liver and kidneys [337]. AgNP excretion was demonstrated by a clinical study, where nanosilver-based dressings were applied to n = 40 patients (3 withdrew) with chronic inflammatory wounds. Half of the subjects displayed elevated Ag levels in the blood serum after 1 month of treatment and no toxicity was detected along with the slow removal of silver from the body [338].

*AgNPs in human blood:* Numerous studies have confirmed the interactions between AgNPs and human blood components such as red blood cells (RBCs), lymphocytes, and leukocytes [339,340,341,342,343,344]. Yet, little is known about the AgNP–RBC interaction mechanisms (e.g., hemolysis, coagulation, and platelet activity) [339]. The inflammatory response within the bloodstream, that is mediated by many cellular components, also contributes to the complexity of these interactions [340]. The PCC properties of AgNPs, such as size, also affect the toxicity to human RBCs. For instance, exposure to 20 µg mL^−1^ of spherical AgNPs of 15 nm in diameter (citrate-coated) induced higher levels of hemolysis (60% hemolysis) and membrane damage when compared to larger AgNPs of 50 nm and 100 nm in diameter (≤12%) [341]. For a specific size of spherical AgNPs (43.9 nm, PVP-coated), structural damage of RBCs was observed to increase with the increase in concentration from 100 µg mL^−1^ to 500 µg mL^−1^ (major membrane damage) [342]. Other cells within the bloodstream can also be damaged by AgNPs. For example, the proliferation and viability of lymphocytes (white blood cells), which help fight disease and infection, were negatively impacted by the exposure to spherical AgNPs (20 nm, PVP- and citrate-coated), and this toxicity was concentration-dependent (10, 20, 30, and 40 µg mL^−1^) [339]. Neutrophils, the most populous cell in the bloodstream, are one of the first lines against pathogens that migrate to sites of inflammation [343]. Exposure to 2, 5, and 20 µg mL^−1^ of spherical AgNPs (20 nm) for 4–20 h activated and increased the population of immunosuppressive neutrophils in circulation [344]. Thus, it was proposed that AgNPs stimulate an anti-inflammatory response by increasing the apoptotic deaths of neutrophils [344].

*Dermal exposure to AgNPs:* The human skin has recently become the subject of numerous toxicity studies due to the widespread use of AgNP-based dermal products. Dermal applications of AgNPs (e.g., wound dressings, wound gels, textiles, and cosmetics—Table 2) are widely used by the public and healthcare providers. Many wound dressings (e.g., Acticoat) contain nanosilver at concentrations of ~50–100 mg mL^−1^ that are above the toxic threshold levels for both fibroblasts and keratinocytes [345]. Skin or immune cells present in topical wounds experience different interactions with and have different tolerance thresholds to AgNPs [346]. For example, human keratinocytes displayed a notably lower viability after 24 and 48 h of exposure to 25 and 50 µg mL^−1^ of AgNPs (30 nm, citrate-coated) [347]. Under identical experimental conditions with citrate-coated AgNPs (30 nm), the viability was significantly lower (≤20%) at concentrations higher than 50 µg mL^−1^, after 24 h [347]. Different responses are also observed in between healthy and damaged tissue samples. For instance, damaged skin exhibited a fivefold higher penetration (2.32 ng cm^−2^) to AgNPs (25 ± 7.1 nm, PVP-coated) than healthy, intact skin (~0.46 ng cm^−2^) [348].

With the pressing dilemma of antibiotic resistance, AgNPs have been explored as a supplement or alternative to antibiotics [150]. Even though there are silver-based products approved by the U.S. FDA (e.g., Silverlon, Aquacel, and Acticoat wound dressings—Table 2), motions have been previously put forward to address the lack of scientific data on the long-term safety of these products [349]. One such proposition, which originated in 1996 and was updated in 2022, recognizes the lack of scientific data on the effectiveness and safety of over-the-counter products containing colloidal silver or silver salts that claim to cure disease. Thus, new products might be mislabeled and require further toxicity research before marketing approval [349]. Potential solutions to the observed toxicity of AgNPs incorporated in consumer products include green synthesis reagents and more biocompatible capping agents of high antibacterial activity.

## 10. Conclusions and Perspectives

The use of silver in its elemental form has been omnipresent in society since historical records began. Before the commercial production of antibiotics, silver compounds were routinely used as treatment for infections and during surgery to prevent infections. Since the 20th century, chemically and biogenically produced nanosilver has been employed not only in medical remedies, but also in commercially available, U.S.-FDA-approved or non-approved products such as clothing, facemasks, and cosmetics. However, the antibacterial mechanistic details of nanosilver and its dependence on the physicochemical properties of nanosilver need further characterization. This review aimed to bridge this gap by focusing on the antibacterial benefits of silver nanoparticles without (AgNPs) and with antibiotic functionalization (AgNP–antibiotic conjugates) against relevant Gram-positive and Gram-negative bacteria. This included ESKAPE pathogens and high-priority antibiotic-resistant bacteria as listed by the World Health Organization.

With over 100 years of nanosilver production, strategies to fabricate AgNPs using chemical reducing agents and bacterial, fungal, or plant extracts are well understood. The antibacterial effects of AgNPs and the subsequent synergy with antibiotics are similarly well established. It is apparent that synergy allows the use of lower antibiotic concentrations, which in turn may mitigate the increase in emergence of antibiotic-resistant microorganisms. In addition, older drugs might be repurposed by conjugating them to AgNPs, as the AgNP–drug constructs can more easily evade microbial defenses. 

Before AgNP-conjugates can become a more common antibacterial treatment, the environmental and health effects of prolonged exposure to Ag and its possible chemical transformations (e.g., from AgNPs to Ag^+^ ionic forms) must also be investigated. Monitoring Ag accumulation in organs such as the skin, liver, kidneys, cornea, and spleen necessitate the development of animal models to establish guidelines for appropriate silver use in order to avoid the negative consequences of overexposure (e.g., Argyria). Certainly, limiting the amounts of antibiotics and silver used to treat infections would be beneficial to the patient and the environment alike. To further lower the dosage and limit the environmental harm of AgNP–antibiotic conjugates, targeted delivery of AgNPs is the next logical step. Targeted delivery is well established in cancer therapies: an antibody, aptamer, peptide, or polysaccharide that is specific to a cell surface receptor is attached to the NP and employed to deliver the NP cargo to only the target cells. Thus, harm to the host and beneficial bacteria can be limited.

AgNPs and AgNP–antibiotic conjugates have great potential as the next generation of antibacterial agents in the post-antibiotic area. Despite solid research on the antibacterial efficacy of AgNPs and their conjugates, there are relatively few U.S.-FDA-approved therapeutics that use this strategy. To realize their true potential, more research is needed. Improving the batch-to-batch reproducibility of AgNP synthesis and the functionalization process, characterizing the PCC properties of AgNPs and AgNP–antibiotic conjugates, and increasing our understanding of how Ag and what forms of Ag affect vital organs are important. Knowledge-based regulatory guidelines are necessary prerequisites before mass production and deployment of AgNP and AgNP–antibiotic conjugates become the go-to antimicrobial therapeutics of the future.

## Figures and Tables

**Table 1:** Overview of the knowledge and applications of silver (Ag) throughout major historical periods. Both household and medical applications are listed, as household use of silver contributed to the foundation of using silver as a therapeutic agent.

	Silver B.C.E. [1,4,7]	Silver Pre-Industrialization [1,7]	Silver during and Post Industrialization [8]
Knowledge	Ag discovered as a metal Ag acknowledged as a food handling tool Ag explored as a basic medicine	Ag acknowledged as a medical treatment First-time correlation of Ag with bacterial treatment	Discovery of bacteria in 1676 Germ theory in 1868 First antimicrobial compound synthesized in a lab, in 1910 First antibiotic (penicillin) discovered in 1928 Ag accepted as a bactericidal agent for infection treatments
Applications	Food and beverage preservation (bulk Ag) Wound care and other medical treatments (e.g., Ag salts and silver leaf)	Silver utensils for food and water consumption (bulk Ag) Wound care (e.g., Ag-based plasters) and other medical treatments (e.g., Ag salts and bulk Ag)	Surgical procedures (e.g., Ag suturing wires) Wound care (e.g., Ag colloids and Ag salts)

**Table 3:** Overview of the chemical, physical, and biological processes for the manufacture of AgNPs: fabrication components, advantages, and disadvantages. The PubMed search words and the associated number (n) of related publications are provided for each type of process.

Processes	Components	Advantages	Disadvantages
**Chemical** [116,123,124] PubMed: “silver nanoparticles chemical fabrication” n = 1459	Solvent Metal precursor Reducing agent(s) Capping agent(s) Functionalization agent(s)—optional	High yield Simple Rapid Low cost Ease of functionalization	Toxic reagents Eco-unfriendly
**Biological** [116,117,120,121,122] PubMed: “silver nanoparticles biological fabrication” n = 940	Metal precursor Solvent Other reagents: plants extracts, bacteria, fungi, enzymes, etc.	Eco-friendly Biocompatible	Wide size distribution Extensive or costly purification
**Physical** [115,116,125] PubMed: “silver nanoparticles physical fabrication” n = 290	Metal source Energy source Solvent	Narrow size distribution Rapid No chemical contamination	Low yield High cost

**Table 4:** Physicochemical (PCC) properties of AgNPs and methods of characterization recommended by the U.S. Environemntal Protection Agency (EPA) [97,116,133,134,135,136,137].

PCC Properties	Characterization Techniques
Size distribution and agglomeration	UV-Vis absorption spectrophotometry, dynamic light scattering (DLS), X-ray diffraction, scanning electron microscopy (SEM), and transmission electron microscopy (TEM)
Shape	SEM, TEM, STM, and atomic force microscopy (AFM)
Surface area and surface-to-volume ratio	TEM and Brunauer–Emmett–Teller measurements
Chemical composition and purity	UV-Vis absorption spectrophotometry, Raman spectroscopy, surface-enhanced Raman spectroscopy (SERS), XPS, flame atomic absorption spectroscopy (FAAS), inductively coupled plasma–optical emission spectroscopy (ICP-OES), ICP–mass spectrometry (ICP-MS), and scanning probe microscopy (SPM)
Surface functionalization	Fourier transform-infrared spectroscopy (FT-IR), Raman spectroscopy, X-ray photoelectron spectroscopy (XPS), thermogravimetric analysis (TGA), nuclear magnetic resonance (NMR), and X-ray diffraction spectroscopy (XRD)
Solubility and surface charge	Solubility tests, zeta potential measurements, electrophoretic mobility, contact angle measurements, hydrophobic interaction chromatography (HIC), atomic force microscopy (AFM), and scanning ion conductance microscopy (SICM)

## Data Availability

All manuscript figures were created with BioRender.
